# HNO-ärztliche Diagnostik und Therapie von Schwindelsyndromen

**DOI:** 10.1007/s00106-025-01592-6

**Published:** 2025-04-07

**Authors:** Leif Erik Walther

**Affiliations:** 1 HNO-Gemeinschaftspraxis, Main-Taunus-Zentrum, 65843 Sulzbach (Taunus), Deutschland; 2 Medizinische Fakultät Mannheim der Universität Heidelberg, Klinik für Hals-Nasen-Ohrenheilkunde, Kopf- und Halschirurgie, Universität Heidelberg, Mannheim, Deutschland

**Keywords:** Periphere Vestibulopathien, Schwindel, Videokopfimpulstest, Morbus Meniere, Lagerungsschwindel, Vestibular failures, Vertigo, Video oculography, Menière’s disease, Positional vertigo

## Abstract

Schwindel ist ein häufiges Leitsymptom in der klinischen Praxis und kann die Lebensqualität stark beeinträchtigen. Mögliche Folgen sind Arbeitsunfähigkeit, eingeschränkte soziale Kontakte, Stürze und sekundäre Erkrankungen. Der HNO-Arzt begegnet sowohl akuten als auch chronischen Schwindelsyndromen im Notfall, in der interdisziplinären Diagnostik und bei HNO-Begutachtungen. Eine strukturierte Anamnese, gezielte Kommunikationstechniken und eine orientierende Untersuchung erleichtern die differenzialdiagnostische Einordnung. Ergänzend ermöglicht eine apparative, objektive Diagnostik („Mapping“ aller 5 Sensoren beidseits) quantitative Ergebnisse über die Funktionsweise der vestibulären Sensoren und Reflexe. Zwar existieren für definierte Krankheitsbilder bereits diagnostische Kriterien und evidenzbasierte Therapien, insgesamt bleibt die Datenlage für viele Erkrankungen jedoch lückenhaft.

**Zusatzmaterial online:**

Die Online-Version dieses Artikels (10.1007/s00106-025-01592-6) enthält weiterführende Literatur.

## Abstract (en)

Vertigo and dizziness are common key symptoms in clinical practice and can significantly impair quality of life. Potential consequences include inability to work, reduced social contacts, falls, and secondary illnesses. Otorhinolaryngologists encounter both acute and chronic dizziness syndromes in emergency settings, during interdisciplinary diagnostic testing, and in ENT expert assessments. A structured medical history, targeted communication techniques, and an orienteering initial examination facilitate differential diagnostic classification. In addition, instrumental, objective diagnostics (“mapping” all five vestibular sensors on both sides) provide quantitative insights into the function of vestibular sensors and reflexes. Although diagnostic criteria and evidence-based treatments exist for certain conditions, the overall data situation remains inadequate for many diseases.

Die Aufgabe des HNO-Arztes besteht in der Diagnostik und Therapie peripherer Schwindelsyndrome (peripherer Vestibulopathien) sowie in der Erfassung differenzialdiagnostischer Aspekte im interdisziplinären Kontext. Diese Rolle ist historisch gewachsen. Mit der Weiterentwicklung der Forschung zu vestibulären Reflexen und modernen objektiven Diagnoseverfahren machte die Schwindeldiagnostik große Fortschritte. Heute erfordert die Behandlung von Schwindelsyndromen einen multidisziplinären Ansatz. Die internationale Bárány-Gesellschaft (für Neurootologie), gegründet 1960, veröffentlicht seit 2009 Kriterien für verschiedene Schwindelsyndrome [1]. Deren Standardisierungsprozess etablierte ein fachübergreifendes Vokabular, das auch international genutzt werden kann. Die interdisziplinäre S2k-Leitlinie „Vestibuläre Funktionsstörungen“ empfiehlt ein interdisziplinäres Management und den rationellen Einsatz von Diagnostik und Therapie [2]. Der Blickwinkel auf „Schwindelsyndrome“ variiert je nach Fachgebiet oder Versorgungsebene, aber auch individuell.

Schwindel ist ein subjektives Symptom und hat einen großen Interpretationsspielraum. „Schwindel“ kann auf periphere oder zentrale Funktionsstörungen zurückgehen, aber auch in Zusammenhang mit emotionalen Konflikten, internistischen oder psychischen Erkrankungen sowie unerwünschten Wirkungen von Medikamenten stehen. Das häufigste Schwindelsyndrom ist derzeit der „funktioneller Schwindel“. Trotz standardisierter Vorgehensweisen und externer Evidenzen sieht sich der behandelnde Arzt heute mit individuellen Faktoren wie Alter, Komorbiditäten und emotionalen Facetten konfrontiert.

Dieser Beitrag stellt häufige und seltene Schwindelsyndrome in Diagnostik und Therapie auf aktuellem Wissensstand dar. Dabei geht es um die „Kunst der Befragung“, die wichtigsten vestibulären Störungen, ihre Kriterien und verfügbaren Evidenzen. Ferner behandelt der Beitrag wichtige praktische Themen, wie den „zervikalen Schwindel“, die HNO-Begutachtung und das Steuern von Kraftfahrzeugen. Tipps und Tricks und ein Fazit sollen praktische Unterstützung bieten.

## Anamnese

### Analyse bei Schwindelsyndromen

#### Verbale Kommunikation

Die Anamnese ist der „Schlüssel“ zum medizinischen Problem bei „Schwindel“. Mit einer analytischen Vorgehensweise („Schwindelanalyse“) kann man sich der Problematik nähern. Zunächst sollte das *zeitliche Auftreten* geklärt werden: akuter (erstmaliger) oder chronischer (permanenter oder rezidivierender) Schwindel. Es muss geklärt werden, ob eine erste, ggf. akute Schwindelepisode oder ein Wiederauftreten vorliegt. Akuter Schwindel (akutes vestibuläres Syndrom, AVS) kann bei einer lebensgefährlichen Erkrankung (akutes zentrales vestibuläres Syndrom, ACVS) auftreten und ähnelt einer akuten peripheren unilateralen Vestibulopathie. Synkopen sprechen für eine kardiologische Ursache und erfordern ein rasches Handeln. Die *Häufigkeit* erlaubt die Zuordnung rezidivierender Beschwerden zu episodischen, anfallsartigen (mit Pausen) oder chronischen Schwindelsyndromen (permanent oder rezidivierend mit beschwerdefreien Intervallen). Der *Charakter* der Empfindung (Drehgefühl, Schwanken, Gangstörung usw.) und Begleitphänomene erleichtern die Differenzierung (episodisches starkes Drehgefühl bei Lagewechsel beim benignen paroxysmalen Lagerungsschwindel, BPLS, das Gefühl, „sich wie auf Wolken zu fühlen“ beim funktionellen Schwindel, ein „Schwarzwerden vor Augen“ beim Aufstehen bei orthostatischer Dysregulation). Viele Vestibulopathien zeigen typische Eigenschaften („Muster“, „Fingerabdruck“). Die *Intensität* und ihr *Verlauf* (Intensitäts-Zeitverlauf) erlauben Rückschlüsse auf die Lebensqualität. Funktionelle Schwindelsyndrome verschlechtern sich meist im Tagesverlauf. Auslösende *Faktoren und Begleitsymptome* (Kopfschmerzen, Hörstörung, Tinnitus) sind ebenfalls wichtig. Kopfschmerzen, eine Photo‑/Phonophobie oder Aura (z. B. „Blitze“, „Kribbeln“) weisen auf eine vestibuläre Migräne hin. Komplexe visuelle Stimuli sind häufig funktionell bedingt [3]. *Medikamente* (Nebenwirkungen, Interaktionen, ototoxische Effekte) und *Begleiterkrankungen* müssen ebenfalls erfasst werden. Schließlich sind *Beeinträchtigungen in Alltag, Beruf *und die *körperliche Belastbarkeit* zu bewerten. Diese Schlüsselfragen ergänzen die Kunst der Gesprächsführung. Denn die Anamnese umfasst mehr als ein informatives Abfragen. Offene und geschlossene Fragen („Multiple-Choice-Fragen“), ebenso nonverbale Aspekte der Gesprächsführung spielen eine Rolle, denn auch Gefühle, Stimmungen und Beziehungen prägen das Patientengespräch maßgeblich.

#### Nonverbale Kommunikation

Zu den nonverbalen Aspekten der Kommunikation zählen Blickkontakt, Gestik, Mimik, Wortwahl, Klang, Gerüche, Haltung und Gang. Sie geben Hinweise auf die emotionale Verfassung, müssen aber verbal abgeglichen werden, um Missverständnisse zu vermeiden. Kommunikation auf Augenhöhe und Perspektivwechsel fördern Vertrauen und helfen, die Situation des Patienten zu verstehen.

Der Patient will Beschwerden äußern und verstanden werden. Zeitdruck ist hinderlich, da individuelle Geschichten und Situationen umfassend erfasst werden müssen. Erfahrung, Intuition und kulturelle Besonderheiten spielen eine Rolle.

Kommunikationshilfen („Yes-Sets“), Metaphern und das „NURSE“-Modell (**N**aming, **U**nderstanding, **R**especting, **S**upporting, **E**xploring) sowie Pausen steigern Empathie. Ein Kommunikationstraining verbessert evidenzbasiert den Umgang mit funktionellen Krankheitsbildern, was positiv wirkt. Funktionelle Störungen erfordern Feingefühl.

Selten gelingt es, sich auf den Patienten „einzuschwingen“ und eine Rührung hervorzurufen, die das Kernproblem offenlegt – ein Zeichen gelungener Kommunikation, Schlüssel zu Diagnose und Therapie.

Persönlichkeitsfaktoren (Neurotizismus, Extraversion, Offenheit, Verträglichkeit, Gewissenhaftigkeit) können erkannt werden. Eine gegenseitige Abstimmung in der Kommunikationssituation sollte auf den 4 Ebenen (Sachinhalt, Selbstkundgabe, Beziehungsaspekt, Appell) übereinstimmen. Das hilft, mit den „Schlüsselfragen“ den Schlüssel passend zu machen (Tab. 1).

**Tab. 1:** Spezifische Fragestellungen bei der Anamnese zu „Schwindel“ im Rahmen der verbalen Kommunikation und Aspekte der nonverbalen Kommunikation

Schlüsselfragen (verbale Aspekte)	Nonverbale Aspekte
Zeitliches Auftreten (Notfall, episodisch oder chronisch)	Wortwahl
Häufigkeit	Stimmeigenschaften (paraverbale Kommunikation)
Charakter (individuelle Wahrnehmung)	Haltung, Bewegungen (Gang, ggf. Hilfsmittel)
Intensität im Zeitverlauf und Entwicklung	Körpersprache (z. B. Mimik und Gestik)
Auslösende Faktoren	Blickkontakt
Medikamente	Emotionale Verfassung (Patient und Arzt)
Begleitsymptome und -erkrankungen	Persönlichkeitsfaktoren
Beeinträchtigung im Alltag und um Beruf	Habitus (unbewusster Gesamteindruck)

### Fragebögen

Mit standardisierten Fragebögen lassen sich Beschwerden analysieren und graduieren. Im deutschen Sprachraum ist der *Dizziness Handicap Inventory (DHI)* nach Jacobsen und Newman etabliert. Die deutsche Version umfasst 25 Fragen zu emotionalen (9), funktionellen (9) und physischen (7) Faktoren mit den Antwortoptionen „Ja“, „Nein“ und „Manchmal“.

- Einsatzbereiche: Erfassung der Lebensqualität bei Schwindel, Überprüfung von Behandlungsresultaten, wissenschaftliche Studien.
- Merkmale: Hohe Genauigkeit und Verlässlichkeit.
- Einschränkungen: Keine klare Zuordnung zu den Teilaspekten in der deutschen Version; interne Konsistenz jedoch praxistauglich.
- Ergebnisse: keine signifikante Korrelation zwischen DHI-Score und Befunden der Vestibularisdiagnostik, auch bei verschiedenen Diagnosegruppen.

Die für den deutschen Sprachgebrauch evaluierte *Vertigo Symptom Scale (VSS)* ist für die Differenzierung psychosomatischer Störungen geeignet, für balancespezifische Aspekte können die deutsche Version des *Activities-specific Balance Confidence Scale (ABC)* oder weitere evaluierte Testverfahren (Testarchiv des Leibnitz-Instituts) genutzt werden.

### Dokumentation von Ereignissen

Bei anfallsartigen, episodischen Schwindelsyndromen wie bei einer vestibulären Migräne, einem M. Menière (MM), einem BPLS usw. sind die Häufigkeit, die Dauer und die Intensität (subjektive Beeinträchtigung) und Begleitsymptome (Kopfschmerzen, Hörstörung, Tinnitus) sowie mögliche Auslöser von Bedeutung. Ein Ereigniskalender ist im Rahmen der diagnostischen Abklärung, nach therapeutischen Interventionen (medikamentöse und chirurgische Interventionen) und zur gutachterlichen Dokumentation und Bewertung empfehlenswert.

### Künstliche Intelligenz

Digitale Kommunikation wird künftig eine große Rolle in bestimmten Bereichen der Medizin einnehmen (z. B. Gesundheits-App Ada, ChatGPT, DeepSeek usw.). Einen Ersatz für das Arzt-Patienten-Gespräch, insbesondere für emotionale Aspekte, stellen Künstliche-Intelligenz(KI)-Varianten noch nicht dar.

### Fazit

Die Anamnese bei Schwindelsyndromen beschränkt sich nicht auf das schematische „Abfragen“ der Beschwerden. Sie erfordert eine Berücksichtigung aktueller Erkenntnisse der Kommunikation. Aspekte der verbalen und nonverbalen Kommunikation sollten bei der Interaktion ebenso berücksichtigt werden, wie das Gesprächssetting (Raum und Zeitfaktor). Fragebögen sind hilfreich bei der Erfassung von Beschwerden. Ein Ereignisfragebogen ist ein hilfreiches Element im Rahmen der Gesamteinschätzung, vor und nach Interventionen. Digitale Kommunikation, wie KI, kann hilfreich sein, Probleme frühzeitig zu identifizieren, ggf. Emotionen zu regulieren, ist jedoch nicht in der Lage, die menschliche Kommunikation zu ersetzen.

## Diagnostik

### Orientierende Diagnostik

#### Nystagmus, Augenbewegungsstörungen

Die Erfassung und Stabilisierung von Blickzielen auf der Stelle des schärfsten Sehens bei bewegten Zielen und Eigenbewegungen erfolgt durch verschiedene funktionelle Systeme: vestibuläres, Sakkaden‑, Fixations-, langsame Blickfolgen‑, Vergenz- und optokinetisches System. Diese Systeme haben zentrale Zentren im Hirnstamm (Augenbewegung) und Großhirn (langsame Blickfolge) [4, 5]. Nach der Anamnese folgt die Untersuchung der Blickmotorik und Nystagmus, um sensorische (periphere) von zentral-vestibulären Störungen sowie der topografischen Einordnung von Störungen der Augenmotorik.

##### Periphere Störungen.

Diese betreffen Augenmuskeln oder versorgende Nerven (z. B. N. oculomotorius) und treten meist einseitig auf („Doppelbilder“).

##### Zentrale Störungen.

Sie betreffen beide Augen („unscharfes Sehen“) und sind oft mit weiteren neurologischen Symptomen (z. B. Sprech‑, Schluck- oder Koordinationsstörungen) assoziiert. Nystagmen (spontan oder provozierbar, z. B. BPLS) sind als Störungen der Blickstabilität aufzufassen. Die differenzierte Analyse von Augenbewegungsstörungen und deren Zuordnung ist Aufgabe des Neurologen, diagnostische Kenntnisse sind jedoch auch für den HNO-Arzt essenziell.

#### Untersuchung der Blickmotorik

In der Neutralposition (Geradeausblick) erfolgt die Untersuchung auf Achsenabweichungen der Augen. Mit dem Abdecktest (abwechselnd monokulär) kann beispielsweise eine vertikale Divergenzstellung auf eine zentrale Läsion hinweisen. Ein horizontaler Spontannystagmus (die Richtung wird nach der schnellen Phase angegeben) deutet meist auf einen peripher-vestibulären Ursprung hin, seltener auf einen zentralen. Die Untersuchung mit der Frenzel-Brille verstärkt die visuelle Wahrnehmung des Untersuchers und macht auch Nystagmen sichtbar, die ohne Hilfsmittel nicht erkennbar sind (visuelle Wahrnehmung ab etwa 4°/s, mit Frenzel-Brille ab 0,5–1°/s). Gleichzeitig verdeutlicht sie die Schlagrichtung, wobei sie eine leichte Abschwächung (Suppression) des Nystagmus bewirken kann.

Für die Analyse von Augenbewegungsstörungen wird der Kopf des Patienten mit einer Hand fixiert. Die Augenbewegungsamplitude wird mit dem Finger der anderen Hand/Gegenstand geprüft (normal: etwa 40 Grad horizontal, 20 Grad vertikal) und anschließend die Augen in den weiteren Blickrichtungen untersucht.

Die wichtigste Methode zur Untersuchung des vestibulären Systems ist der *klinische Kopfimpulstest* (in horizontaler Ebene). Offene Rückstellsakkaden weisen auf eine periphere Vestibulopathie hin, verdeckte Sakkaden sind schwieriger zu identifizieren, können aber durch Erhöhung der Amplitude der Kopfdrehung sichtbar gemacht werden.

Bei der Untersuchung des *Sakkadensystems* (abwechselnde Fixation der Finger des Untersuchers in horizontaler und vertikaler Ebene) sollten sakkadierte Blickfolgebewegungen sowie Hypo- oder Hypermetrien erkannt werden, die für zentrale Störungen sprechen. Sichtbare Sakkaden bei Kopfbewegung in der Horizontalebene deuten auf eine Störung des langsamen Blickfolgesystems hin, wie sie beim CANVAS-Syndrom (**C**erebellar **A**taxia, **N**europathy, **V**estibular **A**reflexia **S**yndrome) auftritt.

Die *Fixation von Blickzielen* wird untersucht, um Veränderungen des Spontannystagmus zu beobachten. Eine Abschwächung der Amplitude und Frequenz bei Fixation eines nahen Ziels (Fixationssuppression) spricht für eine periphere Vestibulopathie. Wenn kein Nystagmus vorhanden ist, kann die Suppression des postrotatorischen Nystagmus mit einem Drehstuhl (drehbarer HNO-Untersuchungsstuhl) oder bei thermischen Tests überprüft werden. Fehlende Suppression deutet auf eine zentrale Störung hin.

*Langsame Blickfolgebewegungen* werden durch Drehung auf dem Drehstuhl geprüft. Der Patient fixiert dabei seine Daumen in Verlängerung der Blickrichtung. Eine fehlende Suppression oder ein vertikaler Spontannystagmus (Downbeat oder Upbeat) weist auf eine zentrale Störung hin. Ein Blickrichtungsnystagmus tritt auf, wenn der Nystagmus in seitlichen Blickpositionen (< 30 Grad) sichtbar wird oder die Schlagrichtung je nach Blickrichtung wechselt. Periodisch alternierender Nystagmus äußert sich durch Oszillopsien und wechselt seine Richtung etwa alle 1–2 min.

Störungen bei der Prüfung der *Vergenz* (Konvergenz und Divergenz) durch Fixation eines nahen (20 cm) und entfernten (2 m) Ziels sind ebenfalls zentralen Ursprungs. Optokinetische Augenbewegungen werden mit einer Streifentrommel untersucht. Unterschiede in Amplitude und Geschwindigkeit des horizontalen oder vertikalen Nystagmus (der in Gegenrichtung zur Trommeldrehung schlägt) können auf eine zentrale Störung hinweisen.

Bei *Provokation durch vestibuläre Reize* (Kopfschütteln, Vibration, Lagerungsmanöver) werden Tonusimbalancen (z. B. Kopfschüttelnystagmus bei unilateralen Vestibulopathien) oder Lage- und Lagerungsnystagmen sichtbar (Tab. 2).

**Tab. 2:** Diagnostische Methoden für die Untersuchung von Augenbewegungsstörungen

Neurophysiologisches System und Funktion	Beispiele für Untersuchungsmethode	Beispiele für Untersuchungsergebnisse
*Vestibulookulärer Reflex:* Stabilisierung des Blickziels	Klinischer Kopfimpulstest Prüfung des horizontalen vestibulookulären Reflexes (hVOR)	Offene (sichtbare) Rückstellsakkade bei peripheren Vestibulopathien
*Sakkadensystem:* Erfassung von Blickzielen und Stabilisierung auf der Fovea	Prüfung horizontaler und vertikaler Sakkaden	Zielungenaue sakkadischen Blicksprünge, (Sakkadendysmetrie) bei Kleinhirnstörungen
*Langsames Blickfolgesystem:* Erfassung, Verfolgen und Stabilisierung des Blickziels	Blickfolgebewegung in den 8 Blickrichtungen	Defizit der Blickhaltefunktion: allseitiger Blickrichtungsnystagmus bei Kleinhirnstörungen
*Vergenzsystem:* Zentriertes Halten des Blickziels auf der Fovea und Tiefensehen (Stereopsis)	Konvergenz- und Divergenzbewegungen	Verlangsamte Konvergenzbewegungen beim M. Parkinson
*Optokinetisches System:* Stabilisierung der visuellen Umwelt als reflektorische Antwort auf großflächige bewegte Blickziele	Streifentrommel (mon- und binokulär) in der horizontalen und vertikalen Blickrichtung	Dissoziierter optokinetischer Nystagmus (internukleäre Ophthalmoplegie)
*Fixation:* Festhalten eines stationären und bewegten Blickziels, auch bei Eigenbewegung	Mon- und binokuläre Fixation beim Geradeausblick mit Abdecktest, Untersuchung in der Blickrichtung	Schielstellungen beim Abdecktest und blickrichtungsabhängige Fixationsinstabilitäten bei zentralen Störungen
*Suppressionsfähigkeit des VOR:* Stabilisierung des Blickziels	Visuelle Suppression eines Nystagmus	Eingeschränkte visuelle Suppression des VOR bei zentralen Störungen

#### Untersuchung von Stand und Gang

Die Untersuchung der *statischen Komponente* (z. B. Romberg-Versuch mit offenen und geschlossenen Augen, Tandemstand usw.) erfasst u. a. Seitenabweichungen in unterschiedlichen Situationen (Augen offen/geschlossen, Seiltänzerstand, Einbeinstand usw.). Bei *dynamischen Tests*, wie Ganguntersuchungen, verschafft man sich einen Eindruck über den Charakter des Gangbilds (Initiierung, Geschwindigkeit, Schrittfrequenz, Koordination, die Kraft, den Muskeltonus, Falltendenzen und die Beendigung des Gangs). Die Untersuchungen eignen sich, um anhand typischer Gangmuster eine grobe Zuordnung vorzunehmen (z. B. hypokinetische Gangstörung beim M. Parkinson, apraktische Störung des Gangs mit Harninkontinenz und demenzieller Entwicklung bei einem Normaldruckhydrozephalus), aber auch für die Beurteilung des Kompensationsgrads peripherer Vestibulopathien. Umfangreichere dynamische normierte Testverfahren, wie z. B. der Mobilitätstest nach Tinetti, der Aufsteh- und Gehtest („Timed-Up-and-Go-Test“), der 6‑Minuten-Gehtetst (6 MGT, „6 Minute Walk Test“, 6 MWT) oder die Bestimmung des De-Morton-Mobilitätsindex (De Morton Mobility Index, DEMMI) sind für den deutschen Sprachraum evaluiert. Diese Testverfahren prüfen auf eine komplexe Art und Weise alltags- und belastungsrelevante Fähigkeiten des Gleichgewichts und eignen sich für die Einschätzung von Gangstörungen, des Sturzrisikos und der vestibulären Kompensation und körperlichen Belastbarkeit aus kardiologischer Sicht. Ausgeprägte Störungen von Stand und Gang (nur mit Hilfe gehen oder stehen können oder die Unfähigkeit zu gehen oder zu stehen) sprechen v. a. im Akutfall immer für zentrale Störungen.

### Apparative Diagnostik

#### Unspezifische und sensorspezifische subjektive und objektive Untersuchungen

Orientierende Tests haben qualitativen Charakter, während apparative Verfahren eine quantitative, objektive Analyse ermöglichen. Mit der videookulographischen Untersuchung (mon- oder binokulär) lassen sich Nystagmen hochauflösend (< 0,5°/s) erfassen, einschließlich Befund- und Videodokumentation.

Die häufigsten Untersuchungen bei Schwindelsyndromen umfassen:

##### Thermische Prüfung.

Mit Wasser (44 und 30°, 50–100 ml), Seitendifferenz ≤ 25 %, Pausen von etwa 2–5 min.

##### Video-Kopfimpulstest (vKIT oder vHIT).

Standardisierte Bedingungen mit fixiertem Ziel (1,5 m Entfernung), Kopfimpulse (15–20°, Dauer 150–200 ms, Geschwindigkeit 150–200°/s). HVOR-Gain etwa ≤ 0,79. Fehlerquellen: zu geringe Drehgeschwindigkeit, lockerer Brillensitz, Wimperntusche oder Reflexionen.

##### Vestibulär evozierte myogene Potenziale (zervikal: cVEMP; okulär: oVEMP).

Untersuchung der überwiegenden Sakkulus- und Utrikulusfunktion (500 Hz, Burst-Reizung 100 dB nHL, 1 ms Rise-Fall-Time, 2 ms Plateau, 50–100 Mittelungen). Ergebnisse: cVEMP-Latenzzeiten 13/23 ms, oVEMP-Latenzen 10/15 ms, Amplitudenverhältnis (AR [%]) = 100 × (größere − kleinere Amplitude) /(größere + kleinere Amplitude). Als Alternative oder für spezielle Erkrankungen eignen sich weitere Stimuli (z. B. Chirps) und Applikationsformen (Knochenleitungsreize).

##### Probleme bei VEMP-Untersuchungen.

Fehlende Vorbereitung (z. B. hohe Hautimpedanz, unzureichende Elektrodenanbringung, Störquellen).

Für apparative Untersuchungen sind Referenzbereiche essenziell, da sie eine objektive und standardisierte Diagnostik ermöglichen.

#### 5-Sensor-Diagnostik und „Mapping“

Die „5-Rezeptoren-Diagnostik“ (besser: „5-Sensor-Diagnostik“) ermöglich eine seiten- und sensorspezifische quantitative Analyse der sensorischen Funktion des Gleichgewichtsorgans. Rückschlüsse auf Erkrankungen können anhand des „Musters“ oder der Rückstellsakkaden nicht gemacht werden. Jedoch ist der Kopfimpulstest sowohl in seiner klinischen (klinischer Kopfimpulstest, cKIT) als auch apparativen Variante (Video-Kopfimpulstest, vKIT oder vHIT) im Rahmen der Differenzialdiagnostik im Notfall unverzichtbar. Am häufigsten findet sich bei akuten unilateralen peripheren Vestibulopathien eine Affektion des horizontalen Bogengangs, der auch eine Dominanz im Vergleich zu den anderen vestibulären Sensoren aufweist.

### Fazit

Orientierende Untersuchungen ermöglichen, im Kontext mit der Anamnese, eine grobe Zuordnung der Beschwerden zu Fachgebieten und Erkrankungen und ermöglichen es, einen „Plan“ für spezifische Untersuchungsmethoden zu entwickeln. Der klinische Kopfimpulstest (Prüfung des horizontalen vestibulookulären Reflexes, hVOR) hat eine Schlüsselstellung in der Diagnostik. Objektive, seiten- und sensorspezifische, quantitative Tests mit der Angabe von evaluierten Referenzbereichen sind bei den apparativen Untersuchungsmethoden zu bevorzugen. Der Video-Kopfimpulstest sollte vor Durchführung der thermischen Prüfung erfolgen.

## Wichtige Schwindelsyndrome

### Schwindelsyndrome als Notfall

#### Akute vestibuläre Schwindelsyndrome

Die Erkennung neurologischer Störungen (Schlaganfall) steht im Vordergrund der Notfalldiagnostik beim HNO-Arzt. Aktuelle Empfehlungen für das Management von akutem Schwindelsyndromen wurden kürzlich von Edlow et al. publiziert. Der Begriff *„akutes vestibuläres Syndrom“ (AVS)* ist danach eine akut auftretende permanente pathologische Wahrnehmung von „Schwindel“ mit Nystagmus, Fallneigung und vegetativen Symptomen.

Eine *akute unilaterale Vestibulopathie (AUVP)* ist meist Ursache einer peripheren Vestibulopathie *(akuten unilateralen peripheren Vestibulopathie, AUPV)* und entsteht seltener infolge einer zentralen Ursache (*akutes zentrales vestibuläres Syndrom, ACVS*).

Ein Aspekt wurde daher früher auch als „Pseudoneuritis“ bezeichnet (z. B. „PICA-Infarkte“, PICA: A. cerebelli inferior posterior). Bei Störungen im hinteren Zirkulationsgebiet (PICA-Affektionen) ist der Kopfimpulstest „negativ“ (meist keine Rückstellsakkaden), bei Störungen im vorderen Zirkulationsbereich („AICA-Stroke“, AICA: A. cerebelli anterior inferior, Pseudolabyrinthitis) sind die Gaindifferenzen im Video-Kopfimpulstest (vKIT, Prüfung des horizontalen vestibulookulären Reflexes, hVOR) sehr gering. Aufgrund der Gefäßversorgung kann bei einer „Pseudolabyrinthitis“ häufiger als bei einer „Pseudoneuritis“ zusätzlich zu „Schwindel“ eine Hörstörung auftreten [6–8]. Die Abgrenzung peripherer (AUPV) von zentralen Störungen (ACVS) ist im Akutstadium durch Einsatz unterschiedlichen Algorithmen mit hoher Sensitivität und Spezifität möglich. Allerdings ist eine sichere Differenzierung von der Erfahrung des Untersuchers abhängig und verbessert sich nach einem Training deutlich.

*Internistische Störungen* (hypertensiver Notfall, Herzrhythmusstörungen, hypoglykämische Krise) sind neben neurologischen Erkrankungen im Notfall ebenfalls eine Herausforderung. Die grobe Erfassung weiterer Parameter (Blutdruck, Puls) sollten Bestandteil der Untersuchung beim HNO-Arzt im Notfall sein.

#### Abgrenzung peripherer Vestibulopathien von zentralen Läsionen

Derzeit valide diagnostische Instrumente sind der **ABCD2-Score**, das **HINTS**-Protokoll [9, 10] (Head Impulse, Nystagmus, Test of Skew), der **vHINTS**-Algorithmus, der **HINTS-PLUS**-Test [11] sowie das **STANDING**-Protokoll [12].

Der *ABCD2-Score* ermöglicht eine 2‑Tages-Risikoermittlung eines Schlaganfalls mittels Punktescore bei einer transitorischen ischämischen Attacke (TIA).

Mit dem *HINTS-Test* ist der erfahrene Untersucher in der Lage, einen Schlaganfall beim Symptom „Schwindel“ mit hoher Sensitivität und Spezifität nachzuweisen. Der HINTS ist innerhalb < 24–48 h sensitiver als eine diffusionsgewichtete Magnetresonanztomographie und ist besser als der ABCD2-Score [9, 10].

Mit dem Konzept des *vHINTS* (Video-Kopfimpulstest, vKIT, anstatt klinischer Kopfimpulstest, cKIT, zur Prüfung des horizontalen vestibulookulären Reflexes, hVOR) konnte die Sensitivität der Erkennung zentraler Störungen im Vergleich zur klinischen Untersuchung um 9 % erhöht werden (Erkennung verdeckter Sakkadden, etwa 13 %).

Für den *HINTS-PLUS*-Algorithmus (Head Impulse Test, d. h. klinischer Kopfimpulstest, zur Prüfung des horizontalen vestibulookulären Reflexes, hVOR), Nystagmus, Test of Skew, PLUS (orientierende Hörprüfung: „finger rub hearing test“, d. h. „Finger hinter dem Kopf aneinander reiben“) ermittelten die Erstautoren eine höhere Sensitivität und Spezifität als beim HINTS-Algorithmus [11]. Zentrale Ursachen für Hörstörungen müssen differenzialdiagnostisch nicht nur bei simultanem „Schwindel“, sondern auch bei einer plötzlichen hochgradigen Hörstörung (hochgradig, beidseits) berücksichtigt werden [12]. Epidemiologische Daten zur Häufigkeit fehlen. In der Gesamtheit der AICA-Infarkte hatten etwa 0,6 % eine akute cochleovestibuläre Störung in Verbindung mit einem ischämischen Schlaganfall im hinteren Kreislauf. Die nachfolgenden Vorgehensweisen werden gegenwärtig empfohlen:

#### HINTS-Plus-Test

[11]

1. *Klinischer Kopfimpulstest:* Ein negativer cKIT (keine Rückstellsakkaden) spricht für einen normalen horizontalen vestibulookulären Reflex (hVOR; zentrale Läsion). Ein positiver cKIT (sichtbare, offene Rückstellsakkade) spricht für eine periphere Vestibulopathie.
2. *Untersuchung der Augen in den Hauptblickpositionen:* Ein Blickrichtungsnystagmus (entgegen der Richtung des Spontannystagmus) spricht für eine zentrale Störung.
3. *Abdecktest:* Bei vertikaler Achsenabweichung der Augen: zentrale Störung.
4. *Finger-Rub-Test* (beim HNO-Arzt apparativer Hörtest!): hochgradige/beidseitige Hörstörung bei zentralen Ursachen selten.

#### STANDING-Algorithmus

Das ***STANDI****N****G***-Protokoll (**S**pon**TA**neous, **N**ystagmus **D**irection, Head **I**mpulse test **G**ait) umfasst 4 Untersuchungsschritte [12]:

1. *Nystagmus*: Untersuchung in den Hauptblickrichtungen, bei Spontannystagmus weiter mit 2. Untersuchung mit der Frenzel-Brille (etwa 5 min) und Ausschluss eines BPLS (falls positiv, ggf. entsprechende Behandlung mit Befreiungsmanövern).
2. *Nystagmusanalyse*: (Bestimmung der Richtung eines *Spontannystagmus*). Ein richtungswechselnder, ein rein rotierender und oder ein vertikaler Nystagmus sprechen für eine zentrale Läsion. Ein richtungsbestimmter Spontannystagmus (unidirektional) wird weiter mit dem klinischen Kopfimpulstest untersucht.
3. *Klinischer Kopfimpulstest*: Liegen sichtbare (offene) Rückstellsakkaden vor, besteht eine periphere Läsion: akute unilaterale (periphere) Vestibulopathie.
4. Untersuchung von *Stand und Gang*: Liegt kein Nystagmus vor (auch kein BPLS) und ist der Patient nicht (oder nur mit Hilfe) in der Lage zu stehen oder zu gehen, spricht das für eine zentrale Störung.

### Fazit

Die genannten Protokolle sind überwiegend von erfahrenen Untersuchern evaluiert; ein Training steigert die diagnostische Sicherheit. Eine zentrale Ursache liegt bei fehlenden Rückstellsakkaden (cKIT), zentralen Augenbewegungsstörungen und ausgeprägten Stand‑/Gangstörungen nahe. Vereinzelt existieren Ausnahmen (z. B. Flokkulusinfarkt mit positivem Kopfimpulstest). Etwa 9 % zentraler Störungen bleiben in der Notfallambulanz unentdeckt. Gottlieb et al. stellten für HINTS und HINTS-Plus eine gute Sensitivität und Spezifität bei erfahrenen Untersuchern fest. Kardiologische Ursachen (Synkopen, Herzrhythmusstörungen, Hypertonie) sind selten.

### Funktioneller Schwindel

#### Funktionelle Störungen

Popkirov u. Weber [13] bezeichnen funktionelle Störungen als „unterbewusst ablaufende, also nicht durch das Bewusstsein steuerbare, jedoch von der Aufmerksamkeit abhängige Verzerrungen der sensiblen Wahrnehmung und Willkürmotorik entlang intuitiver und erlernter Symptomschablonen und Krankheitserwartungen“. Sie „entziehen sich definitionsgemäß einer biomorphologischen Befundsicherung auf Organebene, denn sie entstehen im Rahmen übergeordneter Wahrnehmungs- und Steuerungsprozesse“. Hauptmerkmal ist die Diskrepanz zwischen dem Fehlen eines strukturellen (organischen) Defizits und subjektiven Problemen (Schwindel, Begleitsymptome, Gangstörung) entgegen der eigenen Überzeugung (psychophysische Störung).

#### Bisherige Bezeichnungen

Unter dem Begriff funktioneller Schwindel sind der „phobische Attacken-Schwankschwindel“, die im vorwiegend im englischen Sprachraum entwickelten Bezeichnungen „space motion discomfort“ und „visual vertigo“ bis hin zu neueren Konzepten („chronic subjective vertigo“) zusammengefasst [14–22], sodass eine internationale Krankheitsdefinition entstanden ist (PPPD). Darüber hinaus existieren Begriffe wie der primäre und sekundäre somatoforme Schwindel, deren Konzepte praktisch gut verständlich erschienen [22, 23], die ebenfalls dem Überbegriff „funktioneller Schwindel“ zugeordnet werden müssen. Dieterich et al. [14] fordern für das Verständnis der Problematik funktioneller Störungen und „Schwindel“ die Berücksichtigung der Tatsache, dass strukturelle (vestibuläre), psychiatrische und funktionelle Störungen sowohl unabhängig existieren als auch kombiniert vorkommen können.

#### Definition von „persistent postural-perceptual dizziness“

Funktionelle Schwindelsyndrome kommen nach aktuellen Studien in der Praxis am häufigsten vor. Sie sind noch zahlreicher vor als häufige organisch bedingte Schwindelsyndrome (z. B. BPLS; [14]). Der HNO-Arzt wird täglich damit konfrontiert. Die Bezeichnung „funktioneller Schwindel“ mit seiner definierten Form des *anhaltenden subjektiven Schwankschwindels*, („persistent postural-perceptual dizziness“, PPPD, synonym: 3PD, auf Deutsch: „anhaltender subjektiver Schwankschwindel“) ist gegenwärtig der (Über‑)Begriff für (früher somatoforme, psychogene, psychische usw.) Schwindelsyndrome [15].

Die derzeitigen Diagnosekriterien der Bárány-Gesellschaft [15] lauten:

1. *Episodischer, fluktuierender Schwindel* seit mehr als 3 Monaten mit allmählicher Verschlimmerungstendenz und Beeinträchtigung der Lebensqualität
2. *Verstärkung* der Beschwerden durch Bewegung und (komplexe) visuelle Reize
3. *Triggerung* durch Bewegungsreize, episodische oder chronische, meist periphere Vestibulopathien, psychologische „Stressfaktoren“, neurologische und psychiatrische Erkrankungen
4. *Anhaltende Stressfaktoren *führen zu *körperlichen Symptomen*
5. *Ausschluss einer anderen Erkrankung*.

Nach Habs et al. [16] unterscheidet man 2 Varianten:

#### Primärer und sekundärer PPPD

Beim *primären PPPD* (p-PPPD) ist „Schwindel“ das führende Symptom, und die Erkrankung entwickelt sich *primär* „aus sich selbst heraus“ (z. B. Stress, chronische Erkrankungen, individuell hohe Empfindlichkeit für visuelle Reize, Phobien oder Panikattacken).

Beim *sekundären PPPD* (s-PPPD) gehen episodische oder chronische Vestibulopathien als Auslöser meist *voraus*. Die Erkrankung entwickelt sich *sekundär*.

In der Folge entwickelt sich eine gestörte Wahrnehmung des Gleichgewichts mit körperlichen Symptomen einer „Gleichgewichtsstörung“. Im Normalfall versucht der Organismus, sich an diese temporäre Situation anzupassen (*Adaptation*), so kommt es u. a. zur Aktivierung von Ersatzsystemen, oder es erfolgt eine *Maladaption* (gesteigerte Aufmerksamkeit für visuelle und Bewegungsreize). Persönlichkeitseigenschaften (Vulnerabilität, Selbstbeobachtung) fördern diese Entstehung. Es können sich sekundäre Symptome (Nackensteifigkeit, Phobien, Fallneigung, ängstliche Gangstörungen mit Sturzneigung) herausbilden. Durch eine Behandlung (*Readaptation*) kann dieser Circulus der Maladaptation durchbrochen werden [17].

Der primäre anhaltende subjektive Schwankschwindel ist seltener (etwa 30 %), der Anteil depressiver und Angststörungen (bis zu 25 %) höher als bei der sekundären Form [16]. Habs et al. fanden als häufigste Ursache (als Trigger) für den sekundären PPPD einen BPLS (27 %), eine vestibuläre Migräne (24 %) sowie eine unilaterale Vestibulopathie (15,6 %). Es überwiegt das weibliche Geschlecht, und in etwa 10 % findet sich auch eine vorbestehende affektive Störung (Depression und Angststörung). Die primäre Form hat einen Altersgipfel zwischen dem 25. und 30. Lebensjahr, die sekundäre Form tritt am häufigsten zwischen dem 50. und 55. Lebensjahr auf [16].

##### Beispiel sekundäre Form.

Erfolgreiche Behandlung eines BPLS (Erstmanifestation). Entwicklung einer sekundären Schwindelsymptomatik (anhaltende fluktuierende und sich verschlimmernde Schwindelsensationen ohne Nachweis einer strukturellen Erkrankung) [17]. Der sekundäre PPPD beinhaltet in diesem Fall eine somatische (vorausgehende) und eine funktionelle (nachfolgende) Komponente.

##### Beispiel primäre Form.

Patientin mit Konfliktsituation im Berufsalltag. Schwankender Gang. Sturzneigung. Seit einigen Wochen wechselnder bestehenden Schwankschwindel, eine Wahrnehmung wie im „Nebel“, eine Niedergeschlagenheit, häufig morgens und nach der Arbeit und an Wochenenden. „Schwindel“ ohne objektive Hinweise für eine strukturelle Störung. Konfliktgespräch: Probleme im Rahmen des Berufs. Selbstregulation über Wochen (Trigger: Konflikt) bleibt aus oder ist verzögert (Disstress), es kam zur Maladaptation. Nach einigen Monaten stellt sich die Patientin erneut mit einem anderen Problem vor und berichtet beiläufig, sie habe das Problem durch Selbstreflexion gelöst. (Readaptation).

Lässt sich der auslösende Faktor (z. B. Konfliktsituation) nicht identifizieren, ist die Zuordnung für den HNO-Arzt ohne interdisziplinäre neurologische/psychiatrische Zusammenarbeit nicht einfach.

#### Therapieoptionen

Die Metapher der „Normwertverstellung“ kann behilflich sein, Erklärungsmodelle für den Patienten zu entwickeln. Sie ist ein bildlich gut vorstellbares Argument, mit dem sich die Erkrankung (Maladaptation, Abweichung vom Normwert) und mögliche Therapieansätze begründen lassen (Readaptation, Wiederkehr zum Normwert). („Die verstärkten Beschwerden sind plausibel und verständlich, sie lassen sich erklären und gut behandeln.“). Es existieren physiotherapeutische (vestibuläres Training), psychotherapeutische (kognitive Verhaltenstherapie) und pharmakologische Behandlungsoptionen, die meist multimodal und individualisiert eingesetzt werden. Ziel ist ein „Durchbrechen“ des Circulus der Maladaptation mit Wiederherstellung der Balancekontrolle, einer „Neukalibrierung“ der Sinneswahrnehmung und Wiederherstellung der Selbstbestimmung im täglichen Leben [24]. Tang et al. untersuchten die Kombination von selektiven Serotonin-Wiederaufnahme-Hemmern (SSRI) und stellten positive Effekte bei gleichzeitiger Physiotherapie fest. Es gibt Hinweise für positive Effekte multimodaler Therapiekonzepte, wie die Kombination einer physiotherapeutischen Behandlung und psychotherapeutische Behandlungsmethoden (kognitive Verhaltenstherapie;), Langzeitergebnisse fehlen. Cochrane-Analysen zeigen für pharmakologische und nichtpharmakologische Therapiemethoden noch keine Evidenz [25, 26].

Das Zeitfenster für eine Chronifizierung (per definitionem „3 Monate“) ist schnell geschlossen, bisher war die Langzeitprognose ungünstig. Ursachen sind Schwierigkeiten im Rahmen der Diagnostik, zu wenige Erkenntnisse zur Pathophysiologie, der geringe Evidenzgrad therapeutischer Optionen, der Zeitgeist (Umgang mit psychischen/somatischen Erkrankungen in Deutschland), der geringe Fokus der Fort- und Weiterbildung für diese Erkrankungen und Mängel in den gegenwärtigen Versorgungsstrukturen.

### Benigner paroxysmaler Lagerungsschwindel

#### Otokonien: Anatomie und Pathophysiologie

**Abb. 1:**
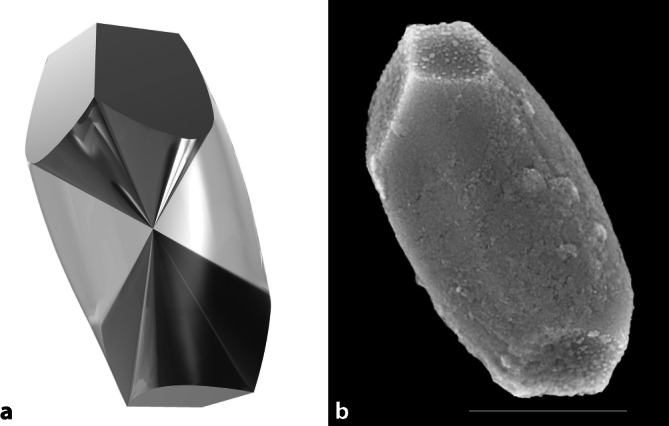
Innere Struktur (**a**) und äußere Form (**b**) einer humanen Otokonie. Erläuterung s. Text. 3‑D-Modell der menschlichen Otokonien mit „belly“ („Bauch“, *hellgrau*) und 3 + 3 „branches“ („Arme“, *dunkelgrau*) mit Treffpunkt aller Strukturen etwa in der Nähe des Symmetriezentrums. Terminale Endflächen: sichtbare Anteile der „branches“, etwa um 60° gegeneinander verdreht. Zum Vergleich in (**b**) das Bild einer intakten, humanen Otokonie. Elektronenmikroskopische Aufnahme im Hochvakuum (HV), 15 kV. Maßstab in **b**: 5 µm. (Bildquelle [89])

Eine ebenfalls sehr häufig im klinischen Alltag anzutreffende Schwindelform ist der benigne paroxysmale Lagerungsschwindel (BPLS). Auslöser sind in die Bogengänge dislozierte, aus dem Utrikulus stammende Partikel (Otokonien) mit anorganischem Material und organischer Matrix. Otokonien sind calcitbasierte (> 90 %) Nanopartikel (Größe etwa 5–30 µm) mit einem geringen Anteil organischer Substanz (< 10 %, [27]; Abb. 1). Das 3‑D-Modell der menschlichen Otokonien zeigt die Struktur von „belly“ („Bauch“) und 3 + 3 „branches“ („Arme“), die sich etwa in der Nähe des Symmetriezentrums treffen. Die terminalen Endflächen stellen die sichtbaren Anteile der „branches“ dar. Sie sind etwa um 60° gegeneinander verdreht.

Bei traumatischen Erkrankungsformen ist eine durch Beschleunigungskräfte bedingte Ablösung von Partikeln aus dem Verbund des Utrikulus sehr wahrscheinlich. Die Erkrankung tritt aber, als Funktion des Alters, auch ohne eine erkennbare Ursache auf. Im fortgeschrittenen Lebensalter ist sie wahrscheinlich mit „degenerativen“ Prozessen assoziiert. Untersuchungen im höheren Lebensalter zeigen schrittweise Veränderungen der anorganischen Struktur, zunächst mit geringen Veränderungen (Porenbildung, Fissuren) und später mit Auswirkungen auf Morphologie und Masse (Calcitreduktion, Frakturen, Partikelbildung), aber auch der organischen Bestandteile (vernetzende Fibrillen), sodass man eine „Degeneration“ (als Summe der im Leben einwirkenden Faktoren) der Otokonien als „natürliches“ Phänomen vermuten kann. Höhere Serumspiegel an Otolin‑1 (ein in Otokonien und der Cochlea befindliches Glykoprotein) werden im Serum bei Patienten mit einem BPLS als „Biomarker“ gedeutet.

Sowohl in den Otolithenorganen als auch im Rahmen von Okklusionsoperationen am Bogengang gelang eine intraoperative Identifizierung von Otokonien mit degenerativen Eigenschaften.

#### Anamnese und Diagnostik beim BPLS

Ein BPLS hat episodischen Charakter und bei der Erstmanifestation (meistens nachts) wird die Erkrankung besonders im höheren Lebensalter als bedrohlich empfunden (Notfall). Nicht selten wird der Bezug zu einem Schlaganfall hergestellt. Kim u. Lee fanden heraus, dass eine Vielzahl aller Notfalleinweisungen in eine Klinik mit „Schwindel“ auf einen BPLS zurückzuführen sind (s. weiterführende Literatur). Die hauptsächlichen Diagnosekriterien lauten: kurzzeitige Schwindelepisoden (etwa 1 min) bei Wechsel der Kopf-Körper-Position und dem Nachweis eines Nystagmus in der Ebene des betroffenen Bogengangs (s. Abschnitt „Diagnostische Kriterien“).

Die Erkrankung betrifft meist singulär einen Bogengang, häufiger das rechte Labyrinth und am häufigsten die hinteren *(etwa 60* *%, pBPLS)* und die horizontalen Bogengänge (*etwa 35–40* *%, hBPLS)*, der vordere Bogengang ist kaum betroffen (da er höher und „über“ den anderen Bogengängen gelegen ist, *aBPLS, etwa 3* *%)*. Am häufigsten sind frei im Endolymphbereich flottierende Partikel (Canalolithiasis), seltener sind Pathomechanismen der Cupulolithiasis und des „canalith jam“, einer „Partikelblockade“ im Endolymphkanal sowie der endolymphatischen Flüssigkeitsbewegung. Canalo- und Cupulolithiasis sind differenzialdiagnostisch meist schwer voneinander zu trennen.

Die Diagnostik erfolgt mittels definierter Lagerungsmanöver (hinterer/vorderer Bogengang: Dix-Hallpike-Manöver, Seitwärtslagerung; horizontaler Bogengang: Supine-Roll-Test, d. h. Kopfbewegung in Rückenlage 90° zu beiden Seiten). Im Fall einer Canalolithiasis (häufigster Fall in der Praxis) zeigt sich nach einer Latenz von wenigen Sekunden ein bogengangspezifischer Nystagmus mit an- und abschwellender Intensität (crescendo-decrescendo) von etwa 1 min.

Eine Cupulolithiasis (pBPLS-cu) ist sehr selten. Hier nutzt man das „halbe Dix-Hallpike-Manöver“, der Kopf wird nicht in die hängende Position gebracht (Nystagmus > 1 min). In der überwiegenden Mehrzahl der Fälle liegt eine Canalolithiasis vor. Beim hBPLS-ca (Canalolithiasis) ist der Nystagmus auf der kranken Seite stärker ausgeprägt. Auch eine höhere Intensität des wahrgenommenen Drehschwindels ist richtungsweisend für das betroffene Ohr. Bei Unklarheiten sollte eine pragmatische Behandlung für beide Seiten erfolgen.

Eine Cupulolithiasis (hBPLS-cu) aufgrund eines „gegenläufigen“ Nystagmus (apogeotroper Nystagmus, zum „oben“ liegenden Ohr) liegt vor, wenn ein stärker schlagender Nystagmus zu beobachten ist, der auch länger (etwa < 2 min) anhalten kann und mit häufig mit starken vegetativen Begleiterscheinungen assoziiert ist. Ein apogeotroper (zum oben liegenden Ohr) schlagender horizontaler Lagerungsnystagmus ist aber seltener und findet sich nur dann, wenn sich die Partikel nahe der Cupula im vorderen Anteil (kurzer Arm) des Bogengangs befinden.

Canalo- und Cupulolithiasis des vorderen Bogengangs (aBPLS) sind Raritäten und zeigen vertikale Nystagmen mit Crescendo-decrescendo-Charakter.

Die visuelle Beobachtung des Nystagmus ist für die sichere Erkennung aufgrund der relativen hohen Geschwindigkeit der langsamen Nystagmusphase bei den verschiedenen Arten des BPLS ausreichend. Die Verwendung elektro- oder videonystagmographischer Diagnostik ist ebenso wie die Nutzung eines Smartphones durch den Untersucher und die Patienten sinnvoll, wenn es um die Objektivierung und Dokumentation geht.

Differenzialdiagnostisch müssen selten vorkommende zentrale Ursachen eines Lage- und Lagerungsschwindels berücksichtigt werden. Länger anhaltende nicht typische, in der Richtung wechselnde oder persistierende Lagerungsnystagmen und/oder zusätzliche neurologische Symptome sprechen für eine zentral-vestibuläre Störung.

Die Nutzung künstlicher neuronaler Netze, die mit vielen Informationen (u. a. Nystagmen) angelernt worden sind, zeigt eine hohe Spezifität in der Erkennung eines BPLS und könnte künftig in der Diagnostik eine Rolle spielen.

#### Physikalische Therapie des hinteren Bogengangs

Sehr gute Daten liegen für das Epley-, das Sémont-, das Gans- und das Brandt-Daroff-Manöver vor ([28, 29]; Tab. 3). Das Brandt-Daroff-Manöver [30] zeigt in einer aktuellen Analyse (systematisches Review) im Vergleich zu den anderen Behandlungsmethoden nur eine geringe Effektivität [29–32].

**Tab. 3:** Aktueller Stand (November 2024) der evidenzbasierten Behandlung des gutartigen Lagerungsschwindels, (Metaanalysen und systematische Reviews) [117, 118, 119, 120, 121, 122, 123, 124, 125, 126, 127]

Bogengang/Erkrankung	Untersuchte Behandlung	Ergebnis	Systematische Reviews und Metaanalysen
Hinterer Bogengang	Epley-Manöver am besten untersucht, Sémont‑, Gans- und Brandt-Daroff-Manöver	Beste Evidenz für Epley-Manöver, Sémont- und Gans-Manöver ähnlich effektiv, Brandt-Daroff-Manöver nur kurzfristiger Effekt	Hilton et al. (2014) Cochrane-Review [117]
Hinterer Bogengang	Betahistin: Auswirkungen auf „Schwindel“ (DHI), Therapieeffekt, Rezidivrate	Besserung von „Schwindel“ (DHI), keine Auswirkungen auf Erkrankung und Rezidivrate	Li et al. (2023) Metaanalyse [118]
Hinterer Bogengang	Sémont‑, Epley- und Brandt-Daroff-Manöver	Therapieeffekt vergleichbar mit Epley- oder Brandt-Daroff-Manöver	Zhang et al. (2017) Metaanalyse [119]
Hinterer Bogengang	Okklusionsoperationen	Effektivität 100 %, aber: Innenohrstörungen	Maas et al. (2020) Metaanalyse [120]
Hinterer Bogengang	Epley- vs. Sémont-Manöver	Epley-Manöver effektiver als Sémont, Rezidivrate identisch	Wang et al. (2013) Metaanalyse [121]
Hinterer Bogengang	Vermeidung von Bewegungen nach Epley-Manöver vs. Epley-Manöver	12–24 h Ruhe sind effektiver als das Epley-Manöver ohne Ruhe	Hunt et al. (2012) [122]
Horizontaler Bogengang	Gufoni-Manöver: sofortige Therapie	Positiver Therapieeffekt	Fu et al. (2020) Metaanalyse [123]
Vorderer Bogengang	Yacovino-Manöver	Effektive Therapiemethode	Anagnostou et al. (2015) systematisches Review [123a]
BPLS	Reduktion der Rezidivrate bei gesichertem Vitamin-D-Mangel	Unterschiedliche Aussagen zur Reduktion der Rezidivrate, die aktuelle Metaanalyse-Studie (2024) [126] spricht dagegen	Yang Z et al. (2021) Jeong et al. (2022) Wood et al. (2024) Metaanalysen [124, 125, 126]
BPLS	Antivertiginosa: beim BPLS (24 h)	Keine Auswirkungen auf die Erkrankung, daher Empfehlung für Lagerungsmanöver	Sharif et al. (2023) Metaanalyse [127]

*BPLS *benigner paroxysmaler Lagerungsschwindel*, DHI *Dizziness Handicap Inventory

Mit dem sog. *Sémont-Plus-Manöver* wurden bessere Ergebnisse ermittelt als mit dem Epley-Manöver (randomisierte placebokontrollierte Studie). Beim Sémont-Plus-Manöver wird der Kopf über das Kopfteil der Untersuchungsliege beim Seitwärtslagerungsmanöver so bewegt (PLUS), dass die Partikel im Bogengang den Scheitelpunkt passieren können [29]. Hochwertige Daten zu Sémont-Plus-Therapie liegen nicht vor.

#### Physikalische Therapie des horizontalen Bogengangs

Beim hBPLS stehen das Barbecue-Manöver (Lempert-Manöver [32]) und das Gufoni-Manöver zur Verfügung. Hochqualitative Daten gibt es für das *Gufoni-Manöver*, welches auch nach einmaliger Durchführung eine gute sofortige Besserung zeigt.

#### Physikalische Therapie des vorderen Bogengangs

Für diese seltenen Formen stehen die Manöver nach Yacovino (besser evaluiert) oder Rahko zur Verfügung.

#### Individualisierte Behandlungsoptionen

##### Therapieresistenz beim pBPLS.

Trotz wiederholter physikalischer Behandlung (Lagerungsmanöver) keine Besserung: Ein mehrfaches forciertes Kopfschütteln vor Durchführung des Epley-Manövers (passiv, 2 Hz, 10 s) führt zu besseren Therapieergebnissen. Im Fall einer *Therapieresistenz beim hBPLS* wird das prolongierte Liegen („shortened/forced prolonged position“) auf einer Seite empfohlen, um eine Mobilisation der Partikel per Schwerkraft zu erleichtern. Eigene Erfahrungen zeigen, dass beim pBPLS ein „doppeltes Sémont-Manöver“ (in Kombination mit vorherigem forciertem Kopfschütteln, 10 × passiv, 2 Hz,) zum Erfolg führt. Der Patient wird forciert 2 × in der Reihenfolge des Sémont-Manövers seitwärts gelagert (5 s in jeder Position warten, in der ersten Position wird der Kopf über 120° nach unten bewegt). In der letzten Position (vor dem Aufrichten) wird so lange gewartet, bis Schwindel (und Nystagmus) vorübergehen.

##### Immobile Patienten.

Bei immobilen Patienten wird die Durchführung des Brandt-Daroff-Manövers (pBPLS) oder das mehrfache Rollen um die eigene Achse in beiden Richtungen auf dem Fußboden (hBPLS) empfohlen (Instruktion und danach Durchführung zu Hause, ggf. mit der Hilfe der Angehörigen).

##### Kein Nystagmus.

Bei typischer Anamnese und fehlendem Nystagmus sollten eine pragmatische Behandlung für alle Bogengänge einmal erfolgen (Fortsetzung einige Tage zu Hause). Hier kann es sich entweder um eine Remission handeln oder um ein Habituationsphänomen.

##### Starke vegetative Erscheinungen.

Es empfiehlt sich eine „Prämedikation“ mit einem Antivertiginosum etwa 30 min vor der Behandlung (z. B. Dimenhydrinat).

##### Die Gesamtsymptomatik passt nicht zur Symptomatik des BPLS.

Da die Entwicklung funktioneller Störungen beim BPLS häufig ist, sollte dies beim Management dieser Erkrankung, v. a. bei Rezidiven und Therapieresistenz, frühzeitig berücksichtigt werden.

Bei stattgehabten *Stürzen oder Sturzgefahr* ist eine Untersuchung auf das Vorliegen eines BPLS sinnvoll, eine sofortige Behandlung verbessert das Sturzrisiko.

##### Mehrere Kanäle betroffen.

Bei traumatischen Störungen oder Therapieresistenz sollte an einen sehr seltenen beidseitigen BPLS (korrespondierende, nichtkorrespondierende Bogengänge) gedacht werden.

#### Medikamentöse Therapie

Eine zusätzliche *Betahistintherapie* zeigte bei Patienten mit einem BPLS des hinteren Bogengangs bessere Ergebnisse als die alleinige Behandlung mit dem Epley-Manöver. Ähnliche Ergebnisse liegen für das Sémont- und das Epley-Manöver vor (Metaanalyse). Aktuelle Metaanalysen zeigen widersprüchliche Ergebnisse bezüglich einer ergänzenden Behandlung mit *Vitamin D* beim BPLS. Ein positiver Effekt auf die Rezidivrate eines BPLS wurde beobachtet, wenn der Referenzbereich im Blut unterschritten ist.

#### Rezidive und Risikofaktoren

Rezidive sind bei allen Arten des BPLS häufig. Die meisten Rezidive treten innerhalb eines Jahres auf, sind unabhängig von der Behandlung und reduzieren sich in der Häufigkeit im Zeitverlauf. Hilton ermittelte eine Rezidivrate von 36 % (48 Monate) nach dem Epley-Manöver. Die Rezidivrate bei Perez et al. betrug 27 %, die meisten Rezidive traten innerhalb von 6 Monaten auf. Chen et al. und Li et al. extrahierten die Rezidivrisikofaktoren weibliches Geschlecht, Alter (≥ 65 Jahre), Hyperlipidämie, Diabetes, Bluthochdruck, Migräne, Vitamin-D-Mangel.

#### Fazit bei der Behandlung des BPLS

Entsprechend der aktuellen Studienlage sollte bei einem pBPLS das Epley-Manöver vor anderen Manövern (Sémont, Gans, Brandt-Daroff) durchgeführt werden. Eine Ruhephase nach dem Epley-Manöver ist effektiv. Bei einem hBPLS sollte zuerst das Gufoni-Manöver erfolgen. Das Yacovino-Manöver stellt eine evidente Therapie des aBPLS dar. Eine zusätzliche, simultane Betahistingabe bessert die Lebensqualität. Vitamin-D-Gaben bei Patienten mit gesichertem Vitamin-D-Mangel senken wahrscheinlich die Rezidivrate (etwa 33 %, 6 Monate), wenn der Serumspiegel erniedrigt ist. Okklusionsoperationen sind eine seltene, aber sichere Therapieoption (Tab. 3). Frei zugängliches Videomaterial zur evidenzbasierten Diagnostik und Therapie findet sich in der Arbeit von Bhattacharyya et al. [33] (s. Infobox 1).

##### Infobox

Maneuvers to Diagnosis and Treat Benign Paroxysmal Positional Vertigo, https://www.youtube.com/watch?v=KLt2LtISPmQ

### M. Menière

#### Diagnostische Kriterien

Die Diagnose eines *definitiven („definite“), klaren, feststehenden M. Menière (MM) *basiert nach den aktuellen Diagnosekriterien [34] auf klinischen Kriterien (episodischer Schwindel, Tinnitus, temporäre Hörminderung [niedrig- bis mittelfrequenter Hörverlust] und/oder Völlegefühl im betroffenen Ohr). Die Episodendauer der Schwindelanfälle (mindestens 2) ist auf einen Zeitraum zwischen 20 min und 12 h begrenzt. Für eine *wahrscheinliche („probable“), vermutliche Menière-Krankheit* wird in einem allgemeineren Konzept ein episodischer Schwindel (mindestens 2 ×) für einen Zeitraum von 20 min bis 24 h gefordert.

Der MM ist eine multifaktorielle Erkrankung, bei der genetische, autoimmunologische und Umweltfaktoren für das Auftreten verantwortlich sind. Eine künftige Subtypisierung wird in der Literatur diskutiert. Im amerikanischen Raum existieren Leitlinien, die die Empfehlungen der Bárány-Gesellschaft aufgreifen. Im deutschen Sprachraum ergab sich für die Akzeptanz der aktuellen Definition des MM [34] nur eine 50%ige Zustimmung [2]. Der Begriff „M. Menière“ wird häufig in der Praxis synonym mit den Begriffen „endolymphatischer Hydrops“ und „hydropische Innenohrerkrankung“ verwendet, was im internationalen Konsens nicht der Fall ist [34].

#### „Endolymphatischer Hydrops“

Ein „endolymphatischer Hydrops“ kann heute auch objektiv, bildmorphologisch mittels Magnetresonanztomographie (MRT) visualisiert werden. Daher wurde versucht, ein eigenständiges Krankheitsbild („hydropische Ohrerkrankung“) zu definieren. Hintergrund ist die Vermutung, dass der endolymphatische Hydrops nicht nur für die vollständige klinische Trias gleichzeitiger Anfälle von Hör- und Vestibularisstörungen verantwortlich ist, sondern auch für andere klinische Erscheinungsformen, wie „vestibuläres“ und „cochleäres Menière-Syndrom“, primäre und sekundäre hydropische Ohrerkrankung, was keinen Einzug in Konsensusdokumente gefunden hat [34]. Fälle mit den Symptomen eines MM können mit und ohne „endolymphatischen Hydrops“ vorkommen. So beschreiben Guajardo-Vergara et al. einen Hydrops auf dem unbeteiligtem Gegenohr im Fall eines gesicherten MM. Van der Lubbe et al. (narratives Review) weisen darauf hin, dass ein Hydrops ein weit verbreitetes Merkmal bei der Menière-Krankheit ist. Ein Hydrops findet sich aber auch zu einem hohen Prozentsatz bei Patienten mit einem Vestibularisschwannom, bei der vestibulären Migräne und bei Gesunden. „Bei Patienten, bei denen ein (sicherer oder wahrscheinlicher) MM diagnostiziert wurde, konnte ein Hydrops seltener mittels MRT diagnostiziert werden als bei der histologischen Bestätigung (82,3 % gegenüber 99,4 %).“ Der endolymphatische Hydrops ist also ein „Marker“ für den MM, erklärt aber nicht allein die Symptomatik und ist nicht allein geeignet, Innenohrsymptome singulär zu begründen. Sun et al. fanden im Rahmen dieser Differenzierung im Zusammenhang mit klinischen Symptomen und anderen Diagnostika bei Patienten mit einer vestibulären Migräne deutlich mehr Fälle mit einem cochleären Hydrops, während beim „MM“ ein Hydrops des Vestibulums häufiger vorkam. Ein Nachweis im Rahmen einer MRT ist sinnvoll, in Ergänzung zu weiteren diagnostischen Hinweisen, um die Diagnose zu stützen. Allerdings hat sich die Durchführung einer Hydrops-MRT in der täglichen diagnostischen Routine in Deutschland noch nicht durchsetzen können. Möglicherweise tragen künftig digitale Datenbanken („Radiomics“, KI-basierte Lernsysteme) dazu bei, den Stellenwert eines Hydrops besser darstellen zu können [35, 36]. Eine große Herausforderung ist darüber hinaus die Differenzierung des MM und der vestibulären Migräne.

#### Differenzierung MM und vestibuläre Migräne

MM und vestibuläre Migräne lassen sich klinisch, v. a. im Anfangsstadium (Normalbefunde), nur schwer unterscheiden. Beide Vestibulopathien sind durch einen episodischen Attackenschwindel charakterisiert, der von Minuten bis hin zu vielen Stunden andauert. Bei beiden Erkrankungen können die Symptome Hörminderung, Ohrdruck, Tinnitus, Kopfschmerzen auftreten. Befunde (Nachweis eines endolymphatischen Hydrops) finden sich ebenfalls bei beiden Erkrankungen. Darüber hinaus ist eine Komorbidität (etwa 15 %) bekannt [34]. Die eindeutige Differenzierung dieses „Zwillingspaars“ wird aufgrund der Symptomüberlappung als „diagnostisches Dilemma“ bezeichnet und ist schwierig, aber im Hinblick auf therapeutische Interventionen notwendig [37, 38].

Unter „Migräne“ (lat. hemicrania, einseitiger Kopfschmerz) versteht man i. Allg. episodisch auftretende, meist einseitige Kopfschmerzattacken: Der Begriff „vestibuläre Migräne“ knüpft daran an, betont jedoch die Kombination von Kopfschmerzen und „Schwindel“. Migräne kommt in der Bevölkerung häufig vor, etwa 12 % der Bevölkerung leiden unter migräneartigen Kopfschmerzen [37, 38].

Die Diagnosekriterien der vestibulären Migräne wurden kürzlich aktualisiert. Bei der vestibulären Migräne sollte in mehr als der Hälfte der Episoden Schwindel mit Kopfschmerzen assoziiert sein. Für die Kopfschmerzen (s. International Classification of Headache Disorders, ICHD‑3, unter A1.6.6 „Vestibular migraine“) sind die Kriterien Einseitigkeit, Pulsation, mittelschwer bis schwere Intensität, Verstärkung bei Bewegung, Photo- und Phonophobie und visuelle Aura definiert worden. Die Symptomatik bildet sich im Frühstadium bei beiden Krankheiten wieder zurück, die Diagnosen ergeben sich für beide Erkrankungen meist erst im Zeitverlauf, in der klinische Praxis erfüllen die meisten Patienten „nur“ das Kriterium einer wahrscheinlichen Diagnose.

Ziel ist es daher, neben der subjektiven Symptomatik, objektive Kriterien mit krankheitstypischen Mustern zu identifizieren, die die Spezifität erhöhen. McGarvie et al. stellten eine Beeinträchtigung des niedrigen Frequenzbereichs des hVOR (thermische Prüfung) fest, während der Mittel-(Drehprüfung) und der Hochfrequenzbereich (Kopfimpulstest) nahezu normale Ergebnisse zeigten. Blödow et al. ermittelten bei 67 % der Patienten mit einem MM eine pathologische thermische Prüfung vs. 22 % der Patienten mit einer vestibulären Migräne. Der hVOR (Video-Kopfimpulstest) zeigte eine Gainreduktion bei 37 % (MM), jedoch nur bei 9 % im Fall einer vestibulären Migräne. Balayeva et al. fanden bei Patienten mit einem MM einen deutlicheren Hörverlust im Tief- und Mitteltonbereich, die cVEMP-Amplituden waren deutlich geringer und der hVOR-Gain niedriger als im Fall einer vestibulären Migräne. Verminderte cVEMP-Amplituden und eine Frequenzverschiebung des Stimulus (1000 Hz) sind Hinweise für einen MM. Im Langzeitverlauf von vielen Jahren vermindern sich die sensorischen Funktionen (Hörvermögen und Gleichgewicht), die Anzahl der Attacken nimmt ab, und eine Beteiligung des Gegenohrs ist häufiger (Tab. 4).

**Tab. 4:** Symptom- und Befundüberlappungen. Sicherer („definite“) und wahrscheinlicher („probable“) M. Menière (MM) oder vestibuläre Migräne (VM) [37, 38, 158, 161, 162, 163, 164, 165]

Merkmal/Symptom	M. Menière	Vestibuläre Migräne
Erkrankungsgipfel	Etwas später, 4.–7. Lebensdekade	Etwas früher (4. Lebensdekade)
Häufigkeit	Deutlich seltener	Relativ häufig (11 % der Population)
Geschlecht und Alter	Geschlecht in etwa gleich verteilt	Deutlich mehr weibliches Geschlecht, bei Kindern
Schwindelepisoden	2 oder mehr Schwindelepisoden	5 oder mehr Attacken
Migränetypische Kopfschmerzen	Etwa 30 %	Etwa 70 %
Aura	Selten	Häufiger
Dauer der Episoden	> 20 min bis 12 h (sicherer MM*) oder 24 h (wahrscheinlicher MM**)	5 min bis 72 h (sichere und wahrscheinliche VM*)
Subjektive Begleitsymptome	Im Vordergrund „Schwindel“ mit Übelkeit und Erbrechen	Häufiger und stärkere Intensität von Übelkeit, Erbrechen, Phonphobie und Photophobie
Cochleäre Symptome	Deutlich stärker ausgeprägte Hörstörung, Völlegefühl, Tinnitus	Eher geringgradig ausgeprägte Hörstörung
Bewegungsabhängigkeit	Geringgradig	Stärker ausgeprägt
Vestibuläre Befunde	Thermische Erregbarkeit häufiger, hVOR-, vKIT- und cVEMP-Beeinträchtigung seltener	Seltener VOR-Störungen, hVOR kaum
Tonaudiometrie	Deutlich messbare tief- bis mittelfrequente Hörstörung	Eher geringgradige Hörstörung
Endolymphatischer Hydrops	Sehr häufig, auch bei anderen Erkrankungen und Gesunden, Hydrops im Vestibulum häufiger	Seltener

*cVEMP *zervikale vestibulär evozierte myogene Potenziale*, hVOR *horizontaler vestibulookulärer Reflex, *vKIT* Video-Kopfimpulstest, *VOR* vestibulookulärer Reflex

#### Fazit

Beide Erkrankungen zeigen Befundüberlappungen (diagnostisches Dilemma), und es kommen Komorbiditäten vor. Der MM ist eine „Innenohrerkrankung“, er zeichnet sich durch häufiger vorkommende und ausgeprägtere Hörstörungen (etwa mehr als 30 dB) und stärkere Beeinträchtigungen des niederfrequenten VOR (thermische Prüfung) aus. Ein zusätzlicher Nachweis einer Beeinträchtigung der überwiegenden Sakkulusfunktion (cVEMP), ggf. mit Verschiebung zu höheren Stimulationsfrequenzen, sowie eine „Hydrops-MRT“ [168] tragen in Zusammenführung von Anamnese und Befunden zu einer Differenzierung bei.

#### Gegenwärtige Evidenzen für Behandlungsmethoden des MM

Es gibt eine Vielzahl von Konsensusdokumenten zur Therapie des MM [2, 34, 169, 170]. Allein im Jahr 2023 erschienen 7 Cochrane-Analysen zu unterschiedlichen Therapiemethoden [39–45]. In 2 Metaanalysen geht es u. a. mit den Auswirkungen auf das Hörvermögen, auch im Rahmen der Chirurgie des Saccus endolymphaticus. Taniguchi et al. ermittelten einen Placeboeffekt bei unterschiedlichen therapeutischen Ansätzen. Weiterhin existiert eine Vielzahl von „Stufenschemata“, die eine potenzielle sensorische Schädigung der Therapiemethode (z. B. „nicht destruktiv“, „destruktiv“) aber nicht die Evidenzniveaus einzelner Therapiemethoden und individuellen Besonderheiten des Patienten berücksichtigt. Beim MM finden sich im fortgeschrittenen Stadium permanente strukturelle (Hörstörung, Tinnitus, periphere Vestibulopathie) und funktionelle Beeinträchtigungen in Form eines PPPD (funktioneller Schwindel, „reaktiver psychogener Schwindel“), die eine gesonderte, individuelle Herangehensweise erfordern (Anfallstherapie, Anfallsprophylaxe, Rehabilitation sensorischer *und* funktioneller Störungen). Die Anfallstherapie ist in ihren Varianten (z. B. Dimenhydrinat, Lorazepam) kaum untersucht.

#### Einflüsse der Ernährung, Drucktherapie

Hussain et al. untersuchten in einer Cochrane-Analyse *Salz‑, Koffein- und Alkoholrestriktionen* ohne den Nachweis eines Effekts. Webster et al. [43] untersuchten in einem umfassenderen Cochrane-Review Auswirkungen von *Ernährung und Lebensstil*. Das einzige in dieser Studie extrahierte Ergebnis war ein Einfluss auf die krankheitsspezifische gesundheitsbezogene Lebensqualität. Bei den Faktoren *Flüssigkeitsaufnahme* und *Schlaf* (Dauer bis zu 2 Jahren) waren keine signifikanten Auswirkungen auf Schwindelsymptome und das Hörvermögen feststellbar.

Für die *Druckbehandlung* des MM extrahierten die Autoren 3 Studien mit insgesamt 238 Patienten [44]. Ein Nutzen konnte nicht bewiesen werden. Diese aktuelle Studie bestätigt die Ergebnisse eines frühere Cochrane-Reviews.

#### Systemische pharmakologische Therapie

Die Evidenz der systemischen Pharmakotherapie (*Betahistin, Diuretika, Antihistaminika, Virostatika, systemische Kortikosteroide*) wird gegenwärtig als „unsicher“ eingeschätzt [45]. Für Betahistin ergab sich in den zugrunde liegenden Studien ein niedriger bis sehr niedriger klinischer Nutzen in einem Zeitraum von 1 Jahr (eine Studie berichtet über eine Besserung innerhalb von 6–12 Monaten, eine Studie über eine Besserung über 12 Monate hinaus, kein Einschluss in die Metaanalyse). Das trifft auch für Diuretika (Isosorbidmononitrat, Amilorid, Hydrochlorothiazid) zu. Die Hochdosistherapie mit Betahistin war Gegenstand kontroverser Diskussionen. Im Ergebnis eines systematischen Reviews ergab sich keine Auswirkung auf die Anfälle. Hochqualitative Studien würden dem Review zufolge derzeit nicht vorliegen. *Für Betahistin* ermittelte ein Cochrane-Review aus dem Jahr 2016 bei unterschiedlichen Ursachen von „Schwindel“ einen positiven, wenn auch geringen Therapieeffekt. Neuere Studien [47, 48] stellten einen signifikanten Vorteil der Kombination von *Cinnarizin und Dimenhydrinat* vs. Betahistin bei Patienten mit „peripherem Schwindel“ fest. Der Nachbeobachtungszeitraum war kurz (4 Wochen), ob Patienten mit einem MM eingeschlossen wurden, geht nicht aus dem Original hervor.

Einen interessanten Ansatz, der zur Dosisreduktion und ggf. zur Wirksamkeitssteigerung von Betahistin beim MM (und anderen peripheren vestibulären Schwindelsyndromen) beitragen könnte, zeigen die Ergebnisse einer Phase-I-Studie mit dem Monoaminooxidase-B-Inhibitor (MAO-B-Hemmer) *Selegilin*, mit dem eine Erhöhung der Bioverfügbarkeit von Betahistin um den Faktor 80–100 erreicht werden kann.

#### Fazit

Faktoren und Maßnahmen der Ernährung, der Lebensqualität oder der Beeinflussung des Mittelohrdrucks sind nicht effektiv, die Therapie mit Betahistin zeigt einen geringen, mittelfristigen Vorteil für die Besserung der Lebensqualität bei „Schwindel“. Ein überzeugender Einfluss auf die Anfälle ist nicht nachgewiesen. Trotzdem sollte eine Therapie mit Betahistin erwogen werden. Das Behandlungskonzept beim MM umfasst Anfallstherapie und -prophylaxe sowie die Rehabilitation der sensorischen und funktionellen Störungen.

#### Intratympanale Applikation von Steroiden

Phillips u. Westerberg haben für eine Dosierung von 4 mg/ml Dexamethason (5 Tage) einen geringen Therapieeffekt ermittelt, der bis zu 24 Monate nachweisbar war (Anfallsprophylaxe) [49]. Webster et al. [41] publizierten 2023 das aktuelle Cochrane-Review (10 Studien mit 952 Teilnehmer, 2–12 mg Dexamethason) und stellten ebenfalls einen geringen Therapieeffekt fest. Am häufigsten wurde Dexamethason eingesetzt. Die Anzahl der Studien mit qualitativ gutem Ansatz ist gering und beträgt im Zeitraum von 2014–2014 („PubMed“) unter den Stichworten „Glucocorticoid Menière“ *n* = 19 (Tab. 5). Einige Studien untersuchten gleichzeitig auch den Effekt der intratympanalen Gentamicinapplikation.

**Tab. 5:** Beispiele für intratympanale Glukokortikoidgaben beim M. Menière in Studien [193, 194, 195, 196, 197]

Medikament	Autoren, Evidenzniveau	Dosierung (mg/ml)	Applikation	Ergebnis
Dexamethason	Sanković-Babić et al. (2014), klinische Studie [193], *n* = 19	4	1 ×	63 % Besserung bis zu einem Jahr
Methylprednisolon	Patel et al. (2016) [194], randomisierte Studie, *n* = 256	62,5	2 × in 2 Wochen	90 % (6 Monate bis 2 Jahre)
Dexamethason	Albu et al. (2016) [195], randomisierte Studie, *n* = 66	4	3 × innerhalb von 3 Tagen	Bis 46,6 % (1 Jahr)
Dexamethason	Molnar et al. (2019) [196], retrospektive Studie, *n* = 105	4	5 × (innerhalb von Monaten?)	12,4 % Hörverbesserung, Follow-up bis 125 Monate
Dexamethason	Phillips et al. (2023) [197], randomisierte Studie, *n* > 1000	12 + Poloxamer	1–2 × in 4 Wochen	90 % bis zu 24 Monate

Phillips et al. ermittelten den Effekt der einmaligen Gabe von 12 mg Dexamethason (plus thermosensitives Poloxamer) in 3 Doppelblindstudien nach einmaliger intratympanaler Injektion. Eine statistisch signifikante Reduktion (im Vergleich zu Placebo) von „Schwindel“ ergab sich in einer Studie (Endpunkt 3 Monate). Alle Studien ergaben eine Reduktion von „Schwindelanfällen“. Der Effekt intratympanaler Gele (auch Hyaluronsäure) als Additivum ist nach Brotto u. Greccio [50] unzureichend untersucht. Cao et al. [51] fanden in ihrer Metaanalyse einen leichten Vorteil für Methylprednisolon gegenüber Dexamethason. In der aktuellen Leitlinie [2] wird eine Dexamethason-Injektion (4 mg) an 3 aufeinanderfolgenden Tagen ([44]; Tab. 5) empfohlen.

#### Intratympanale Gentamicinapplikation

Die Anzahl der Studien mit Metanalysen, systematischen Reviews, Randomisierung (Doppelblindstudie) und klinischen Trials ist gering und beträgt im Zeitraum von 2014 bis 2024 unter den Stichworten „Gentamicin und Menière“ (PubMed) *n* = 23. Das aktuelle Cochrane-Review (2023) mit eingeschlossenen Studien und *n* = 137 Patienten ergab keine nachweisbare Evidenz [42].

Patel et al. (s. weiterführende Literatur) untersuchten in einer randomisierten kontrollierten Studie die Gabe von Methylprednisolon und Gentamicin (je 30 Patienten) und stellten innerhalb von 18–24 Monate nach der Injektion eine 90%ige vs. 87%ige Kontrolle episodischer Schwindelattacken fest. Die Patienten erhielten 2 intratympanale Gentamicin-Injektionen (62,5 mg/ml) und je 2 Injektionen Methylprednisolon (40 mg/ml) innerhalb von 2 Wochen. Bei etwa 20 mg Methylprednisolon ergibt sich ein Dexamethasonäquivalent von etwa 3 mg pro Injektion. Cao et al. (s. weiterführende Literatur) analysierten 9 Studien mit *n* = 314 Patienten (3–28 Monate Nachbeobachtung). Gentamicin war am effektivsten, Kortikoide und Gentamicin hatten einen vergleichbaren Langzeiteffekt. Yaz et al. [52] stellten eine geringe Überlegenheit von Gentamicin gegenüber Glukokortikoiden fest. Das systematische Review von van Esch (s. weiterführende Literatur; 19 randomisierte Doppelblindstudien) ermittelte eine geringe Evidenz für die intratympanale Anwendung von Gentamicin und Glukokortikoiden. Hao et al. (s. weiterführende Literatur) extrahierten 10 Studien mit *n* = 455 Patienten (Gentamicin und Dexamethason), es ergab sich ein guter Effekt mit geringen Unterschieden zwischen Gentamicin und Dexamethason hinsichtlich der Kontrolle von „Schwindel“. De Amesti et al. (s. weiterführende Literatur) fanden in ihrer Metaanalyse nur eine sehr geringe Evidenz für die Kontrolle von Schwindel durch Gentamicin. In publizierten Protokollen werden 267 mg/ml Gentamicin (bis zu 4 Injektionen in wöchentlichem Abstand) oder etwa 40 mg/ml (bis zu 5 Injektionen in 14-tägigem Abstand) verwendet und unterschiedliche Endpunkte (subjektive Reduktion der Anfälle, hVOR-Reduktion im Video-Kopfimpulstest und eine Hörschwellenänderung von mehr als 10 dB im Tonaudiogramm) genutzt.

Die Bezeichnung einer „Ausschaltung“ des Gleichgewichtsorgans mit Gentamicin ist nach Lange et al. (s. weiterführende Literatur) und neueren Untersuchungen nicht mehr zutreffend, da eine geringe Dosierung und Einmalgabe ebenfalls als effektiv angesehen werden, um die episodischen Schwindelattacken mit Erhalt oder sehr geringer Auswirkung auf das Hörvermögen zu kontrollieren. Das bestätigt auch eine Metaanalyse von Salt et al. (s. weiterführende Literatur). Über weitere Therapiemethoden (z. B. Lidocain), wie die „Labyrinthanästhesie“, gibt es nur vereinzelt Untersuchungen, in denen keine Hörbeeinträchtigung ermittelt worden ist [53, 54].

#### Chirurgische Behandlungsoptionen

In älteren kontrollierten Studien zeigten sich geringfügige Therapieeffekte [55, 56]. Pullens et al. [57] ermittelten in ihrem Cochrane-Review keine evidenten Behandlungsergebnisse für die Saccusoperation. Es existieren unterschiedliche Varianten der Eingriffe am Saccus endolymphaticus (Saccusexposition, Shuntoperation). In einer aktuellen Arbeit von Szott et al. [179] (s. weiterführende Literatur, Metaanalyse) ergab sich, dass ein Eingriff am Saccus endolymphaticus vorteilhaft sein kann, da positive Effekte auf die Lebensqualität (Schwindel) nachgewiesen werden können, während eine negative Auswirkung auf den durchschnittlichen Reintonhörverlust und das Sprachverständnis festgestellt wurde. Lyu et al. (s. weiterführende Literatur) stellten einen positiven Effekt nach dem Verschluss aller 3 Bogengänge und zusätzlicher Glukokortikoidgabe fest. De la Cruz et al. vergleichen die translabyrinthäre Vestibularisneurektomie und die transmastoidale Labyrinthektomie und fanden einen Vorteil für die Vestibularisneurektomie [58]. Die Neurektomie des N. vestibularis zeigt einen langfristigen Effekt bei einseitigen Erkrankungen [59]. Eine Neurektomie bessert jedoch nicht die cochleären Symptome eines MM.

#### Hörrehabilitation beim MM

Eine Rehabilitation des Hörvermögens ist bei einer einseitigen oder beidseitigen Verschlechterung des Hörvermögens indiziert (Hörgeräte, Cochleaimplantation). Berardino et al. (s. weiterführende Literatur) und Selleck et al. (s. weiterführende Literatur) stellten in ihren Metaanalysen eine Verbesserung der Lebensqualität fest, insbesondere eine Hörverbesserung, unabhängig von der Krankheitsdauer, der Lokalisation (ein- oder beidseitige Erkrankung), dem Alter, früheren therapeutischen Verfahren und dem Aktivitätsstadium der Erkrankung. Auch kombinierte Therapiemethoden sind beschrieben, wie die Durchführung einer Labyrinthektomie mit Neurektomie des N. vestibularis (ggf. mit simultaner Cochleaimplantation).

#### Fazit

Nach der gegenwärtigen Studienlage gehen *intratympanale Glukokortikoidgaben* beim MM mit einer zeitweiligen Hörverbesserung und Anfallsreduktion einher. Der positive Effekt bei Schwindel und Hörstörungen kann etwa 6 Monate bis 2 Jahre anhalten. Mit Dexamethason kann die höchste Konzentration/Wirkstärke (das 6‑Fache von Prednison oder Methylprednisolon) und die längste Wirkdauer (3,5–72 h) erreicht werden. Derzeit können Methylprednisolon (100 mg/ml) und Dexamethason (10 mg/ml) in Deutschland genutzt werden. Eine Dauerbehandlung mit *Betahistin* (begleitend) bessert die Lebensqualität bei „Schwindel“ (ohne Einfluss auf die Schwindelepisoden).

*Gentamicin* zeigt ebenfalls einen mittelfristigen Therapieeffekt und ist auch bei Einmalgabe (keine „destruktive“ Methode unter dem Aspekt der Hörfunktion und keine „Ausschaltung“ unter dem Aspekt der sensorischen Gleichgewichtsfunktion) effektiv, bei Mehrfachgaben ist das Risiko der Hörverschlechterung hoch.

*Chirurgische Behandlungsoptionen* zeigen ebenfalls positive Effekte. Auch in Abhängigkeit vom Leidensdruck (Ereigniskalender) ist beim MM ein individualisierter Therapieansatz zu empfehlen. Eine „schematisierte“ Behandlung scheint unter diesem Aspekt nicht mehr zeitgemäß.

### Akute unilaterale periphere Vestibulopathie

#### Diagnostische Kriterien, Varianten und Verlauf

Für die akute unilaterale periphere Vestibulopathie (früher z. B. „Neuritis vestibularis“) gibt es neue diagnostische Kriterien [60]. Die Erkrankung bezieht sich auf einseitige periphere sensorische/neurale Schäden mit akutem Beginn (Spontannystagmus, Fallneigung, vegetative Symptome); eine Hörstörung sowie eine zentral-vestibuläre Erkrankung sind ausgeschlossen.

Die „5-Sensor-Diagnostik“ ermöglicht eine Differenzierung solcher Störungen, und gegenwärtig lassen sich bei einer akuten unilateralen Vestibulopathie 4 Typen extrahieren:

1. die vollständige Beteiligung aller 5 Sensoren (Vollbild),
2. die Beteiligung der Sensoren des oberen Asts des N. vestibularis (obere Form),
3. die Beteiligung der Sensoren des unteren Asts des N. vestibularis (untere Form),
4. die Beteiligung des horizontalen Bogengangs (isoliert, ampulläre Form).

Die Affektion des N. vestibularis superior ist am häufigsten anzutreffen. Raritäten stellen isolierte Störungen der sensorischen Funktion der Bogengänge und der Otolithenorgane dar.

Eine Restitutio ad integrum wird in etwa 50 % der Fälle beschrieben. Teil- und Vollschäden können persistieren. Je nach dem Grad der vestibulären Kompensation erfolgt bei fehlender oder teilweiser Erholung eine Besserung der Beschwerden im Zeitverlauf.

#### Therapie der akuten unilateralen Vestibulopathie

Die Ursache der akuten unilateralen Vestibulopathie ist unbekannt. Virale, autoimmunologische und vaskuläre Ursachen werden diskutiert. Hillier u. McDonnel fanden in ihren Cochrane-Reviews, dass die physikalische Therapie eine sichere und wirksame Behandlung einer einseitigen peripheren Vestibularisfunktionsstörung ist [61]. Das gleichgewichtserhaltende System benötigt einen frühzeitigen „Input“, um zentralnervöse Prozesse der Wiederherstellung (Readaptation, Habituation) zu aktivieren (s. Abschnitt Vestibuläre Kompensation). Aktuelle Studien zeigen, dass eine frühzeitige Behandlung vorteilhaft ist. Auch Lacour et al. beobachteten durch frühzeitige physikalische Rehabilitation eine frühere Rückbildung eines Spontannystagmus als ohne Therapie (s. weiterführende Literatur). Hidayati und Kim et al. stellten positive Auswirkungen der Kortikosteroidbehandlung (kurzzeitige Effekte) auf die Erholung der Sensorfunktion und langfristige Effekte für eine vestibuläre Trainingsbehandlung fest [62, 63].

Betahistin und die Kombination von Cinnarizin und Dimenhydrinat wirken sich kurzfristig (etwa 4 Wochen) positiv auf die Lebensqualität aus [64, 65].

Strupp et al. beschreiben in ihrer Studie eine Wirksamkeit für Methylprednisolon (100 mg), nicht aber für ein Virostatikum (Valaciclovir). Die Behandlung (i.v.) kann bis zu einem Monat nach Behandlungsbeginn effektiv sein, um jedoch eine Anflutung im Innenohr zu erreichen, sind wahrscheinlich etwa 250 mg Prednisolon erforderlich. Eine kombinierte, frühzeitige Rehabilitation plus Kortikosteroide ist effektiver als eine alleinige Kortikosteroidgabe [66].

Im Akutstadium dominieren vegetative Symptome mit Übelkeit und Erbrechen (Elektrolytverlust). Sedativa und Benzodiazepine sollten nur für kurze Zeit verabreicht werden (1–3 Tage). Sie wirken sich negativ auf die Diagnostik (mögliche Beeinflussung des VOR) und die vestibuläre Kompensation aus.

#### Fazit

Eine frühzeitige physikalische Rehabilitation (Trainingselemente: Gleichgewicht, Koordination, Blickübungen, Kraft und Ausdauer) ist auf dem höchsten Evidenzniveau langfristig effektiv. Derzeit gibt es Hinweise für positive Effekte einer frühzeitigen Behandlung mit Kortikosteroiden (etwa mindestens 250 mg Prednisolonäquivalent) in Kombination mit einer vestibulären Trainingstherapie. Auf das Risiko der Entstehung funktioneller Schwindelsyndrome sollte im Therapieverlauf geachtet werden.

#### Vestibuläre Kompensation

In der Akutphase einer unilateralen Vestibulopathie entsteht immer eine Tonusimbalance (Fallneigung, Spontannystagmus, belastungsabhängiger „Schwindel“), die sich jedoch spontan im Zeitverlauf zunächst spontan bessert. Unter vestibulärer Kompensation versteht man einen zentralen schädigungs-, zeit- und altersabhängigen Prozess mit der Eigenschaft einer individuell unterschiedlichen funktionellen Verbesserung *statischer* (z. B. Standstabilität) und *dynamischer Defizite* (z. B. Blickstabilisierung, Gang). Die statische Komponente ist i. d. R. nach wenigen Wochen (bis zu 3 Monate nach dem Akutereignis) funktionell gut kompensiert. Die Kompensation der dynamischen Komponente hingegen nimmt länger, ggf. einige Wochen oder Monate, in Anspruch. Die Mechanismen sind bis heute nicht vollständig verstanden. Lacour et al. (s. weiterführende Literatur) fanden Unterschiede der vestibulären Kompensation bei unterschiedlichen Schädigungsmechanismen, wie neurogene Neurektomie, labyrinthäre (z. B. Labyrinthektomie) und toxische Schädigungen (z. B. durch Gentamicin) im Tierversuch. Yazdanshenas et al. (s. weiterführende Literatur) bestätigten diese Ergebnisse (ebenfalls im Tierversuch) und stellten eine zügigere vestibuläre Kompensation bei einer ototoxischen Behandlung vs. Labyrinthektomie fest.

Sensorische Störungen können zu Beeinträchtigungen der kognitiven Funktionen führen, insbesondere der visuell-räumlichen Wahrnehmung (Hippocampusreduktion).

Für die objektive Abschätzung des Grads der vestibulären Kompensation gibt es Klassifikationen und objektive Bewertungsmaßstäbe, die sich auch für die Begutachtung eignen. Die Einschätzung des Grads der vestibulären Kompensation sollte mithilfe der apparativen Erfassung von Spontan-(Elektro‑, Videonystagmographie, wenn verfügbar: rotatorischer Test) und Provokationsnystagmen (Kopfschüttelnystagmus in der Ebene der horizontalen Bogengänge, 30-mal, 45° zur Seite, 2 Hz) erfolgen. Das Fehlen von Spontan- und Provokationsnystagmus spricht nach der gegenwärtigen Lehrmeinung für einen hohen vestibulären Kompensationsgrad. Diskrepanzen sprechen für eine zusätzliche, meist funktionelle Störung. Die Einschätzung von Stand und Gang erfolgt mit posturographischen Methoden und evaluierten Tests. Störungen des hVOR sind besonders dann von Bedeutung, wenn offene Rückstellsakkaden im Video-Kopfimpulstest persistieren, die zu einer Beeinträchtigung der dynamischen Sehschärfe führen. Da bei höheren Beeinträchtigungen, z. B. schnellen Kopfbewegungen, nur der VOR in der Lage ist, die Blickachse bei intaktem Visus zu stabilisieren, eignen sich andere Systeme nicht als „Ersatzsysteme“. Beeinträchtigungen des hVOR mit offenen Rückstellsakkaden im Video-Kopfimpulstest wirken sich daher besonders stark auf das Befinden aus. In der Akutphase findet sich auch eine Beeinträchtigung des hVOR der gesunden Seite im Sinne einer geringen Reduktion des kontralateralen Gain, auch bis unter den Normalbereich im Video-Kopfimpulstest (hVOR; „Cerebellar-Shutdown/Clampdown-Theorie“: Inhibition der neuronalen Aktivität in den Vestibulariskerngebieten durch das Kleinhirn). Pharmaka und Genussmittel können vestibuläre Kompensation wahrscheinlich negativ (Alkohol, Antihistaminika, Benzodiazepine oder Neuroleptika) oder positiv (Koffein, Glukokortikoide) beeinflussen.

### Bilaterale Vestibulopathie

Die anamnestischen Hinweise für eine bilaterale Vestibulopathie sind nicht immer eindeutig. Die Patienten klagen über einen bewegungsabhängigen „Schwindel“, der sich im Dunkeln verstärken kann (Unsicherheit, Fallneigung, Gangstörungen, Balanceprobleme bei unebenem Untergrund, Stürze und Oszillopsien). Gerade im höheren Lebensalter sollte nach solchen Störungen gefahndet werden, da sie eine Hauptursache für Stürze darstellen können. Die diagnostischen Kriterien der Bárány-Gesellschaft fordern eine Störung des hVOR beidseits (hVOR-Gain < 0,6) und/oder eine Beeinträchtigung der thermischen Erregbarkeit [67]. Die Beschwerden variieren nach dem Muster, der Schwere der Beeinträchtigung des VOR, dem Grad der Beeinträchtigung der Blickstabilisierung (offene oder verdeckte Rückstellsakkaden beim hVOR mit der Korrelation zu Oszillopsien) und dem Lebensalter. Ist die Störung bereits zur Geburt vorhanden oder frühkindlich erworben, sind die Beschwerden deutlich geringer ausgeprägt als bei erworbenen Störungen.

In der Praxis lassen sich viele Muster bilateraler sensorischer Beeinträchtigungen (Voll- oder Teilschäden) aller 5 Sensoren auf beiden Seiten beobachten, auch solche, welche nicht die genannten Kriterien erfüllen. (z. B. einzeln oder kombiniert, korrespondierend oder nichtkorrespondierend). Murofushi et al. verglichen die Symptomatik einer bilateralen Vestibulopathie der hinteren Bogengänge (Teilschädigung) mit denen einer hVOR-Beeinträchtigung (s. weiterführende Literatur). Die Symptomatik war deutlich geringer ausgeprägt, Oszillopsien waren im Dunkeln nicht vorhanden. Das ist ein Hinweis für die Dominanz des hVOR. Tarnutzer et al. untersuchten retrospektiv *n* = 109 Patienten mit einer bilateralen Vestibulopathie und fanden am häufigsten eine Beteiligung der hinteren Bogengänge, gefolgt vom hVOR und den vorderen Bogengängen [68]. Teilschäden manifestieren sich in der Akutphase immer mit geringeren Beschwerden als Vollschäden.

In etwa 2 Drittel der Fälle findet sich keine Ursache [252], in einigen Fällen lassen sich aber im Rahmen der interdisziplinären Diagnostik (Neurologie, Innere Medizin) ätiologische Zusammenhänge identifizieren, z. B. traumatische Ursachen, das Cogan-Syndrom (Autoimmunerkrankung mit Augen- und Ohrbeteiligung), das CANVAS-Syndrom (Begleitsymptome u.a. chronischer Hustenreiz, Schluckstörungen), eine vestibulotoxische Medikation (Aminoglykosidtherapie), bilaterale Vestibularisschwannome (Neurofibromatose Typ 2), Schädel-Hirn-Traumen, Meningitis, bilateraler MM [69]. Selten sind Fallbeschreibungen bei einer Otosyphilis oder einem systemischen Lupus erythematodes, neurodegenerativen Erkrankungen (Machado-Joseph-Erkrankung), komplexen Mechanismen mit Neoplasien und zytostatischer Behandlung und/oder Medikamentennebenwirkungen und genetischen Ursachen (bei familiärer Häufung) in der Literatur vorhanden. Moyaert et al. stellten als häufigste Ursache genetische Veränderungen fest (Mutationen im COCH-Gen, Cochlin, Chromosom 14q12–q13; [70, 71]).

In Anhängigkeit vom Lebensalter und vom Status sowie der Funktionsweise der vestibulären Rezeptoren, insbesondere auch des Hoch-(Video-Kopfimpulstest) und Niederfrequenzbereichs (thermische Prüfung) des hVOR, sind individualisierte Therapiemethoden indiziert. Die Konzepte ähneln den Rehabilitationsmethoden bei unilateralen Vestibulopathien.

Bei schweren Beeinträchtigungen (hochgradige hVOR-Beeinträchtigung im Video-Kopfimpulstest), Sturzgefahr oder stattgehabten Stürzen und höherem Lebensalter (mit Komorbiditäten) empfiehlt sich ein kontinuierliches Training (assistierte Physiotherapie bei Sturzgefahr) mit den Trainingselementen für die Muskelkraft, Balance, Koordination usw.

Galvanische Stimulationsmethoden oder biofeedbackbasierte Systeme tragen neben unter Assistenz durchgeführten physiotherapeutischen Trainingsprogrammen als mögliche konservative Therapiemethoden zu einer Verbesserung der Lebensqualität und Sturzprävention bei [72–74].

Die Entwicklung von und Rehabilitation mit „vestibular implants“ wird in der Literatur beschrieben . Es wird über eine Besserung der Lebensqualität, insbesondere die Reduktion von Stürzen, berichtet. Ausführliche Erläuterungen zur Funktionsweise finden sich in der Arbeit von Chow et al. (s. weiterführende Literatur)

Lebensqualität, Trainingszustand und die Entwicklung der sensorischen Parameter („5-Sensor-Diagnostik“) sollten regelmäßig beobachtet und dokumentiert werden.

### Schwindelsyndrome im höheren Lebensalter

#### Veränderungen der vestibulären Sensoren und diagnostische Erfassung

Für die vestibulären Haarzellen Typ 1 und Typ 2 ist eine Reduktion in den Crista- (> 40 %) und Maculaorganen (etwa 20–25 %) ab der 7. Lebensdekade nachweisbar. Unspezifische Altersveränderungen sind die Akkumulation von Lipofuszin (wahrscheinlich nicht abbaubares quervernetztes Aggregat, bestehend aus oxidierten Protein- und Lipidclustern; [75]).

Wenige Studien zu Veränderungen des hVOR zeigen, dass diese Funktion nach bisherigen Erkenntnissen bis ins hohe Lebensalter relativ stabil bleibt. McGarvie et al. fanden eine geringfügige Gainreduktion bei sehr hohen Kopfgeschwindigkeiten für den hVOR (etwa ab 200°/s). Pogson et al. ermittelten mit zunehmendem Alter eine höhere Variabilität der vertikalen Bogengänge, außerdem war eine Zunahme der Frequenzen, der Amplituden und der Spitzengeschwindigkeit von Sakkaden feststellbar. Mallison u. Longridge stellten keine Veränderungen des niedrigfrequenten hVOR (thermische Prüfung) im Alter fest.

#### Veränderungen in den Otolithenorganen und Auswirkungen auf die Otolithenfunktion

Im Bereich der Otolithenorgane kommt als Funktion des Alters zu einer Reduktion der Anzahl und der Masse der Haarzellen, im Sakkulus deutlicher als im Utrikulus, sowie zu strukturellen Veränderungen der Otokonien (s. auch Kapitel „Benigner paroxysmaler Lagerungsschwindel“) sowie zu zentralen Veränderungen der neuralen Elemente im Sinne einer Reduktion [75]. Rosa et al. ermittelten in einer neueren Studie, dass oVEMP (als ein Indikator für die präsente Otolithenfunktionsstörung) häufiger pathologische Veränderungen aufweisen. Pauwels et al. fanden eine erhöhte Sturzrate und Gangveränderungen bei Patienten mit einem BPLS.

Wenige Studien berichten über eine messbare Verminderung der Otolithenfunktion mit zunehmendem Alter, so erhöhen sich die VEMP-Schwellen, und die Amplituden werden kleiner.

Aber auch Körperbewegungen müssen ausgeglichen werden, um die Objektwahrnehmung bei linearen Bewegungen (translatorisch) zu realisieren. Der Beitrag der Otolithenorgane hierzu ist bekannt (translationaler vestibulookulärer Reflex, tVOR). Der Beitrag der Otolithenorgane und des tVOR ist u. a. aber über die Perzeption von Linearbeschleunigungen, der Schwerkraft und von Kopfkippungen bekannt Der tVOR trägt zu einer Minimierung der Bewegung des Netzhautbilds zwischen Objekten bei, die in unterschiedlichen Tiefenebenen liegen, um die Bewegungsparallaxeninformationen zu optimieren. Liao et al. vermuten einen Zusammenhang zwischen reduzierter Otolithenfunktion, tVOR-Beeinträchtigung und Stürzen. 3‑D-Analysen der Bewegungsmuster könnten künftig in der Diagnostik hilfreich sein.

#### Presbyvestibulopathie

Als Presbyvestibulopathie bezeichnet man nach den Kriterien der Bárány-Gesellschaft [76] derzeit eine altersassoziierte (> 60 Jahre) leichte Beeinträchtigung des hVOR im Sinne einer bilateralen Vestibulopathie mit Gainwerten von 0,6–0,8, die aufgrund physiologischer Veränderungen (individuell unterschiedliche physiologische Beeinträchtigung des VOR im peripheren und zentralen Bereich) entsteht. Bei der altersassoziierten Erkrankung finden sich auch eine Störung der Standstabilität, Gangstörungen und Stürze. Als „Alterungsphänotyp“ wird eine allmähliche Verschlechterung mehrerer physiologischer Systeme im höheren Lebensalter bezeichnet. In vielen Fällen ist „Schwindel“ im höheren Lebensalter ein Summationseffekt von physiologischen Veränderungen, Begleiterkrankungen, Medikamentennebenwirkungen und deren Interaktionen. Es wird vermutet, dass die vestibuläre Funktion im Zusammenwirken mit anderen (sensomotorischen, visuellen usw.) altersabhängig abgewandelt wird. „Altersschwindel“ oder „Presbyvertigo“ ist derzeit nicht in den Krankheitsklassifikationen definiert.

### Herpes zoster oticus

Beim Zoster oticus ist „Schwindel“ ein häufiges Symptom. Bei der klinischen Symptomatik (Fazialisparese: etwa 87 %; Neuritis vestibularis: etwa 77 %; Hörstörungen: etwa 36 %, meist pancochleäre und cochleobasale Muster; andere Hirnnervenbeteiligungen etwa < 5 %) imponieren Ohrenschmerzen [77, 78]. Pathognomonisch für die seltene Erkrankung (1:20.000) sind herpetiforme Effloreszenzen im Bereich der Ohrmuschel (Herpes: „kriechen“, zoster „Gürtel“), die nach einer Windpockeninfektion (Latenz in Spinal- und Hirnnervenganglien) im Rahmen einer „Immunsuppression“ reaktiviert werden (Gen 63). Die Prophylaxe mit einem Varizellenimpfstoff bei Kindern (in Deutschland 2 ×, 11.–14. und 15.–23. Monat, bis zum 17. Lebensjahr möglich) kann seit 2004 in Anspruch genommen werden, Zosterimpfstoffe sind in Deutschland seit 2013 und 2018 auf dem Markt, die Ständige Impfkommission (STIKO) empfiehlt ausschließlich die Verwendung des Varizella-Zoster-Virus-Glykoprotein-E-Antigens obligatorisch ab dem 60. Lebensjahr und bei immunsuppressiver Ausgangslage ab dem 50. Lebensjahr. Eine mittelfristige Wirksamkeit des Zosterimpfstoffs sowie eine Reduktion der Infektionsfälle (Windpocken, Zoster) sind bereits epidemiologisch messbar. Eine frühe Therapie mit einem Virostatikum (je nach Verlauf p.o. oder i.v.), eine Schmerzbehandlung sowie eine topische virostatische Therapie werden in der aktuellen Leitlinie bei Zoster oticus (bis zur Abheilung der Effloreszenzen, etwa 1 Woche) empfohlen. Komplikationen (neurologische Komplikationen, Augenbeteiligung, postherpetische Neuralgie, vaskuläre Ereignisse wie Schlaganfall und Herzinfarkt gehäuft 3 Monate bzw. 1 Jahr nach der Erkrankung) müssen berücksichtigt werden. Obwohl die höchste Evidenz weder einen Vorteil für Kortikosteroide noch Glukokortikoide ergibt, spricht die Datenlage für eine solche Behandlung (Leitlinie) bei jedem Zoster im Kopf-Hals-Bereich. Für die Behandlung der Vestibularisstörung (schlechtere Prognose) sind auch Therapieempfehlungen der unilateralen Vestibulopathie zu berücksichtigen.

### Syndrome eines dritten mobilen Fensters

Eine Dehiszenz des oberen Bogengangs („superior canal dehiscence syndrome“) ist ein seltenes, zu den Drittfenstersyndromen zählendes Krankheitsbild, welches sich in erster Linie mit audiovestibulären Symptomen äußert. Dabei muss die knöcherne Bedeckung des Bogengangs nicht vollständig fehlen („near dehiscence“, Ausdünnung der Knochenschale des betroffenen Bogengangs). Die diagnostischen Kriterien der Bárány-Gesellschaft fordern für eine sichere Diagnose eine Symptomatik über wenigstens ein Jahr: Knochenleitungshyperakusis, geräusch- und druckinduzierbarer Schwindel oder Oszillopsien über den Zeitraum der Stimulation und pulsatiler Tinnitus. Die Ursachen sind nicht eindeutig aufgeklärt, traumatische Ereignisse, eine Störung der postnatalen Knochenentwicklung, eine familiäre Häufung sowie genetische Ursachen werden vermutet [80].

Es wird angenommen, dass es bei einer Anregung über den Luftschall zu einem *Entweichen* von Schallenergie über das dritte Fenster kommt, also eine veränderte Innenohrmechanik (normalerweise via ovales und rundes Fenster) vorliegt. Knochenleitungsreize hingegen führen dazu, dass Schallwellen aus dem Schädel via drittes Fenster *ins Innenohr gelangen* (Hyperakusis). Die Symptomatik ist in Tab. 6 dargestellt.

**Tab. 6:** Audiologische und vestibuläre Symptome und Befunde bei Dehiszenzsyndrom des oberen Bogengangs [80, 295, 296]

Audiologische Symptome und Befunde	Vestibuläre Symptome und Befunde
Autophonie (verstärkte oder verzerrte Wahrnehmung der eignen Stimme, Nachhall), Hyperakusis (akustische Phänomene beim Kauen, Laufen, Bewegen der Augen, verstärkte Wahrnehmung von Darmgeräuschen), Ohrdruck, temporärer Tinnitus	„Schwindel“ bei Druckerhöhung im äußeren Gehörgang, beim Valsalva-Versuch, beim Pressen, Heben, Schnäuzen oder überschwelligen akustischen Reizen (Tullio-Phänomen)
Weber-Versuch lateralisiert ins betroffene Ohr, Schalleitungsstörung von Typ der elastischen Versteifung – Schallleitungsstörung im Tieftonbereich, negative Knochenleitungsschwellen (< 0 dB im Tieftonbereich als Korrelat für die Autophonie)	Verminderte Schwellen für cVEMP und erhöhte Amplituden der oVEMP, vorübergehender Nystagmus (ampullofugal), exzitatorisch, bei Erhöhung des Drucks im äußeren Gehörgang (Hennebert-Zeichen) oder bei überschwelligen Luftleitungsreizen oder ampullopetal (inhibitorisch) bei Erhöhung des intrakraniellen Drucks

*cVEMP *zervikale vestibulär evozierte myogene Potenziale, *oVEMP* okuläre vestibulär evozierte myogene Potenziale

Im Rahmen der Diagnostik sollte eine Computertomographie mit Dünnschichtung < 1 mm erfolgen, ggf. eine sekundäre 3‑D-Rekonstruktion aller 3 Bogengänge beider Seiten. Ward et al. machen darauf aufmerksam, dass Dehiszenzen in einigen Fällen auch gefunden werden, jedoch keine entsprechende Symptomatik vorhanden ist; sie diskutieren eine protektive Rolle der Dura mater in solchen Fällen. Differenzialdiagnostische Überschneidungen sind für die vestibuläre Migräne (Komorbiditäten möglich) und den MM (bei Tinnitus) beschrieben [80].

Ziel der Behandlung ist eine Verminderung oder Beseitigung der Mobilität des dritten Fensters bei starker Beeinträchtigung der Lebensqualität durch Vermeidung oder chirurgische Behandlung (transmastoidale oder transkranielle Obliteration des betroffenen Bogengangs, „Plugging“) oder eine Wiederherstellung der Kontinuität des dehiszenten Bogengangs, z. B. mit Knochenmehl, Knochenchips und Faszie („Resurfacing“). Einige Autoren beschreiben eine Symptomlinderung durch eine alleinige Verstärkung des runden Fensters, die Methode ergab aber in einer Studie von Succar et al. keinen Langzeiteffekt. Bunne et al. empfehlen den Eingriff nur als Alternative, wenn der individuelle Gesundheitszustand des Betroffenen keinen Eingriff am Bogengang erlaubt und wenn ein Therapiewunsch besteht

### Vestibularisparoxysmie

Die diagnostischen Kriterien der „Vestibularisparoxysmie“ (vestibuläre, selten audiologische Symptome aufgrund ephaptischer Entladungen im proximalen Teil des VIII. Hirnnervs) sind seit 2016 definiert [81]. Leitsymptom sind sich wiederholende, von der Art her identische kurzzeitige Schwindelepisoden. Die *wahrscheinliche Erkrankung* umfasst *5 Anfälle von Dreh- oder Nichtdrehschwindel, einer Dauer von <* *5* *min*, ein spontanes Auftreten oder Triggerung durch bestimmte Kopfbewegungen sowie eine stereotype Phänomenologie. Für die *gesicherte* Erkrankung werden *10 Attacken mit einer Dauer von <* *1* *min* gefordert (stereotype Phänomenologie), ein Ansprechen auf Carbamazepin oder Oxacarbazepin [81].

Cowen et al. kommen in einer Metaanalyse zu dem Ergebnis, dass nur ein *neurovaskulärer Kontakt* (und nicht allein eine in etwa 25 % vorkommende „Gefäßschlinge“) in Kombination mit den beschriebenen Symptomen, nicht aber ohne diese die Kriterien einer Vestibularisparoxysmie erfüllen. Weitere hochqualitative Studien liegen nicht vor. Hochauflösende MRT-Techniken können bei der Diagnosestellung einen wichtigen Beitrag leisten.

Fallberichte existieren über einen intermittierenden Tinnitus und „Schwindel“ (richtungswechselnder Nystagmus) oder eine neurovaskuläre Kompression, die durch eine A. subarcuata verursacht wurde.

Für die Therapie existieren kaum hochwertige Daten. In einer randomisierten Studie wurde ein Vorteil für die Behandlung mit Carbamazepin und Betahistin ermittelt. Eine placebokontrollierte Studie von Bayer et al. ergab einen positiven Behandlungseffekt für das Carbamazepin-Derivat Oxcarbazepin [82].

### Traumatische, infektiöse und toxisch bedingte Schwindelsyndrome

#### Traumatisch induzierte Perilymphfistel

Rupturen der Fenstermembranen werden „vermutet“, wenn sich Schwindelsyndrome in Kombination mit meist ausgeprägten Hörstörungen und ggf. Tinnitus präsentieren. Um eine traumatisch induzierte Perilymphfistel mit vestibulärer Beteiligung zu objektivieren, ist der Nachweis eines lageinduzierten Nystagmus auf der Seite der Läsion zu fordern („Fensterfistelsymptom“ [83]). Der objektive Nachweis gelingt durch sichtbare traumatische Veränderungen (intraoperative Frakturlinie, Gehörknöchelchen- und Membranrupturen) sowie Luftansammlungen im Bereich des Innenohrs bei der hochauflösenden computertomographischen Darstellung („Pneumolabyrinth“). Eine Analyse der abfließenden Perilymphflüssigkeit (z. B. Beta-Trace-Protein) sichert die Diagnose zusätzlich mit einer hohen Wahrscheinlichkeit. Die Ursachen einer traumatischen Membranruptur sind vielfältig und individuell (z. B. Schädelfrakturen, traumatische Gehörknöchelchenluxationen oder -dislokationen, pressorische Ereignisse, Tauchgang) [84]. Klassifikationskriterien fehlen bisher. Rawal et al. berichten über eine erfolgreiche endoskopische Behandlung bei einer Fallserie mit traumatisch bedingten Perilymphfisteln. Nakajima et al. fordern eine frühzeitige Intervention im Hinblick auf den Erhalt der cochleären und vestibulären Funktion [85]. Die vestibuläre Funktion zeigte bei frühzeitiger Intervention eine bessere Prognose als die Hörstörungen.

#### Traumatische Labyrinthschädigungen

Traumatische Schäden des Innenohrs entstehen durch direkte Gewalteinwirkung auf den Schädel, wobei die otische Kapsel (Innenohr, knöchernes Labyrinth) betroffen sein kann, oder selten durch pressorische Ereignisse. Das Labyrinth ist bei Querfrakturen häufiger betroffen als bei Längsfrakturen. Darüber hinaus wird „Schwindel“ häufig im Zusammenhang mit Distorsionsverletzungen der Halswirbelsäule mit oder ohne direkten Schädelkontakt beklagt, außerdem wird der HNO-Arzt mit „Schwindel“ bei Dezelerationstraumata mit resultierenden Scherverletzungen konfrontiert [86] („traumatic brain injury“, TBI, isolierte Hirnverletzung). Schädelprellungen sind unfallmechanisch (kausal) mit einer geringeren Krafteinwirkung auf das Innenohr assoziiert, sie können mit und ohne Verletzungen auftreten. Bei Schädelprellungen (aus neurologischer Sicht) sind keine funktionellen oder strukturellen Schäden im Hirnbereich nachweisbar.

Bei strukturellen Verletzungen des Innenohrs treten die Symptome, wie „Schwindel“, Hörstörungen, Tinnitus oder Ohrdruck, meist sofort auf; in bestimmten Fällen (z. B. BPLS, Bogengangdehiszenz) bei traumatischen Schwindelsyndromen auch nach einem geringgradigem Trauma mit einem längeren freien Intervall. Diskrepanzen zwischen der Schwere der Gewalteinwirkung und dem Vorliegen objektiver Verletzungszeichen sind häufig Gegenstand juristischer Auseinandersetzungen [86]. In diesem Zusammenhang fallen auch häufig die historischen Begriffe „Commotio labyrinthi“ oder „Contusio labyrinthi“. Daher sind eine lückenlose Dokumentation sowie die Beurteilung der Art und Schwere des Schädelkontakts von großer Bedeutung. Für die Beurteilung der Gewalteinwirkung sind objektive Verletzungszeichen (z. B. Hämatome, Wunden, Frakturen) und eine neurologische Einschätzung wesentlich.

Die neurologischen Definitionen verwenden ähnliche Begriffe, wie „Commotio cerebri“ („Gehirnerschütterung“) und „Contusio cerebri“ („Hirnprellung“), die jedoch unfallmechanisch nicht auf traumatische Schädigungen des Gleichgewichtsorgans übertragen werden können. Der Begriff „Schädel-Hirn-Trauma“ (SHT) wird nur dann verwendet, wenn es zu einer zumindest vorübergehenden Funktionsstörung des Gehirns oder substanziellen Hirnverletzungen (infolge eines „traumatic brain injury“, TBI) gekommen ist. Schädelprellungen ohne strukturelle Schädigungen sind davon abzugrenzen.

Die historischen Begriffe „Commotio labyrinthi“ und „Contusio labyrinthi“ werden trotz ihrer fehlenden Grundlage in internationalen Krankheitsklassifikationen aus medizinischer oder juristischer Perspektive immer wieder verwendet. Vereinzelt werden sogar Subklassifikationen („Commotio labyrinthi vestibularis“ und „Commotio labyrinthi cochlearis“) vorgeschlagen, die sich auf temporäre oder permanente Gesundheitsstörungen (z. B. Otolithenfunktionsstörungen) beziehen. Weitere Begriffe wie „Bagatelltrauma“ oder „postkommotionelles Syndrom“ [86] oder „stumpfes Schädeltraumata“ [86] sind in der Lehrbuchliteratur erwähnt. Hier besteht Forschungs- und Klassifikationsbedarf. Stattdessen ist zu empfehlen, objektiv nachweisbare, strukturelle Gesundheitsschäden (nach ICD) infolge eines Ereignisses (Schädeltrauma) zu benennen, z. B. (akute) unilaterale Vestibulopathie (H81.3); infolge einer Schädelbasisfraktur: S02.4. Der frühere Begriff der „Mikrofrakturen“ ist heute nicht mehr zeitgemäß, vielmehr lassen sich Frakturen der otischen Kapsel (keine Kallusbildung) noch nach Jahren mittels Dünnschicht-Computertomogramm objektiv erfassen.

Die Therapieprinzipien orientieren sich an der Art der Schädigung: Bei strukturellen Defekten können chirurgische Eingriffe, eine absteigende Kortikoidbehandlung oder ggf. eine Antibiotikagabe erforderlich sein. Evidenzen hierzu sind nicht vorhanden.

#### Infektiös bedingte Schwindelsyndrome

Ein peripher-vestibulärer „Schwindel“ im Rahmen einer grippalen Infektion findet sich am häufigsten mit dem Nachweis einer Mittelohrfunktionsstörung (exsudativer Tubenmittelohrkatarrh, „toxisches Exsudat“). Während Hörstörungen (meist Innenohrbeteiligung im Hochtonbereich) relativ häufig sind und eine gute Prognose haben, gilt „Schwindel“ mit nachweisbarem Nystagmus (hVOR-Schädigung) als ein „Alarmzeichen“ einer fortschreitenden Innenohrstörung (zeitnahe Parazentese, ggf. mit Paukendrainage, symptomatische Behandlung, Glukokortikoidgabe, ggf. antibiotische Therapie). Virusinfektionen können im Rahmen der Infektion mit neurotropen Viren beobachtet werden (Mumps, Maserninfektion), aber auch im Rahmen einer Borreliose oder bei anderen bakteriellen Infektionen (akute und chronische Otitis media). Hochqualitative Daten zu Therapien sind nicht verfügbar.

#### Ototoxisch bedingte Schwindelsyndrome

In sehr hoher Dosierung (2–3 g/Tag) sind Schwindelsyndrome im Zusammenhang mit Salicylat beschrieben („Aspirin-“ oder „Salicylat-Ototoxizität“; [87]). Häufiger ist in der Praxis eine Behandlung mit Aminoglykosiden (Gentamicin; meist bilaterale Vestibulopathie), der vestibulotoxische Effekt tritt hier mit einer Verzögerung (etwa 2 Wochen ein) und ist irreversibel (i.v.-Gabe). Die intratympanale Applikation scheint jedoch hinsichtlich der Schwindelepisoden zumindest teilweise „reversibel“ zu sein. Für die Applikation von Aminoglykosiden existieren Grenzdosen.

### Zervikogener Schwindel

Der Zusammenhang zwischen Halswirbelsäule (HWS) und Schwindel wird oft diskutiert, ist aber international nicht einheitlich definiert [88]. Begleitsymptome sind Nackenschmerzen, Steifigkeit, Kopfschmerzen und eine Bewegungsabhängigkeit. Es sind keine zielführenden diagnostischen Tests verfügbar. Als objektiver Beweis für einen zervikogenen Schwindel („cervical dizziness“) wird die Auslösung von Schwindel und Nystagmus gefordert.

Das Positionspapier „Cervical dizziness“ [88] der Bárány-Society enthält u. a. folgende Ursachenhypothesen:

- Zufallshypothese: häufiges gemeinsames Auftreten von Nackenschmerzen und Schwindel
- Neurovaskulär: Irritation des sympathischen Plexus
- Somatosensorisch: Störung propriozeptiver Afferenzen
- Trigeminus: Schwindel durch Nackenschmerzen via N. trigeminus
- Synkope: Irritation des Karotissinus, Bradykardie, Hypotonie, Präsynkopen
- Migräne: Nackenschmerzen und Schwindel als Migräne oder Subtypen

Hinsichtlich der Behandlung von Gelenkfehlstellungen (über eine Beeinflussung der Arthrokinematik) gibt es 2 hochwertige randomisierte kontrollierte Studien, die über eine Besserung von Schmerzen und „Schwindel“ berichten.

Nervenverbindungen (Reflexwege) existieren zwischen zervikalen und zentralen Regionen jedoch nicht unmittelbar zwischen sensorischen (peripheren vestibulären) und zervikalen Gebieten.

Der wissenschaftliche Beweis eines „cervical dizziness“ (Schwindel, der von der HWS ausgeht) steht noch aus.

### Steuern von Kraftfahrzeugen bei Schwindelsyndromen

#### Kriterien in Deutschland

Laut „Begutachtungsleitlinien zur Kraftfahreignung“ gilt: „Wer unter ständigen, anfallsartigen Störungen des Gleichgewichts leidet, ist nicht fahrtüchtig“ [89]. Der HNO-Arzt sollte eine Empfehlung aussprechen und dokumentieren. Eine Eigenverantwortung gemäß § 1 StVO besteht weiterhin [90]. In der Regel liegt eine situative Beeinträchtigung (der *Fahrtüchtigkeit*) vor. Dieser Begriff beschreibt die Fähigkeit einer Person, ein Fahrzeug zu einem konkreten Zeitpunkt (über einen gewissen Zeitraum) sicher zu führen. Diese Einschränkung kann auch die generelle *Fahreignung* (generelle Fähigkeit zum Führen/Steuern eines Kfz) betreffen. 

#### Kriterien in Europa

Einige Autoren, wie Huppert und Brandt, kritisieren die Begutachtungsleitlinien [89] aus neurologischer Perspektive als „zu kategorisch“ und „zu streng“ und plädieren für eine Überarbeitung [91, 92]. Sie argumentieren, dass die Dauer der geforderten Anfallsfreiheit bei vergleichbaren Erkrankungen kürzer sein könnte als in den Kriterien der Bundesanstalt für Straßenwesen (BASt). Besonders bei vestibulärer Migräne und bilateraler Vestibulopathie sei die Verkehrsgefährdung nach Huppert und Brandt deutlich geringer als bisher angenommen [91]. Andere Studien belegen das. McDougall et al. wiesen nach, dass Patienten mit bilateraler Vestibulopathie überraschend gute Leistungen beim Fahren erbringen [93]. Bisher gibt es auch keine belastbaren Untersuchungen, die belegen, dass Patienten mit „Schwindel“ ein erhöhtes Unfallrisiko haben (s. auch [94]). Eine einheitliche Rechtslage in Europa zu diesem Thema existiert derzeit nicht.

Etwa 20 % aller zugelassenen Medikamente in Deutschland haben verkehrsrelevante Nebenwirkungen. Geschätzt 25 % der Verkehrsunfälle sollen im Zusammenhang mit Medikamenten stehen [95]. Cannabis erhöht das Unfallrisiko bis auf das 3‑Fache. Die Blutalkoholgrenzen variieren international; Alkohol verursacht in hohen Konzentrationen vestibuläre Beeinträchtigungen (bilaterale Vestibulopathie) und zentrale Effekte [96, 97].

### HNO-Begutachtung bei Schwindelsyndromen

Häufig stellt sich in verschiedenen Rechtsgebieten die Frage, ob „Schwindel“ und seine Auswirkungen im täglichen Leben und im Beruf auf strukturelle Schädigungen im HNO-Fachbereich (periphere Vestibulopathien) zurückzuführen ist. Aktuelle Vorschläge berücksichtigen objektive Untersuchungsmethoden für episodische und permanente, ein- und beidseitige periphere Vestibulopathien im Zusammenhang der Beurteilung von Grad der Behinderung (GdB)/Grad der Schädigungsfolgen (GdS)/Minderung der Erwerbsfähigkeit (MdE). Damit kann auf der Grundlage des aktuellen wissenschaftlichen Erkenntnisstands mit sehr hoher Wahrscheinlichkeit ermittelt werden, ob eine periphere Vestibulopathie vorliegt, welches *Ausmaß* diese hat (Voll- oder Teilschaden), welche Sensoren betroffen sind (*Art der Schädigung*), und welche *Eigenschaften* vorliegen (z. B. offene Rückstellsakkaden mit Beeinträchtigung der Blickstabilisierung). Die Bestimmung von Kompensationsgrad und Organschadengrad führt zur Schätzung/Festsetzung von MdE/GdB/GdS bei peripheren Vestibulopathien [98–101].

## Fazit für die Praxis

- „Schwindel“ ist ein häufiges Symptom, das eine **interdisziplinäre differenzialdiagnostische Einordnung** erfordert.
- Eine **sorgfältige Diagnostik** ist der „Schlüssel“ (Anamnese, orientierende und apparative Untersuchungen mit internationalen Diagnosekriterien).
- Die Erkennung **zentraler** **vestibulärer** **Störungen** kann heute mit hoher diagnostischer Sicherheit erfolgen.
- Für die einzelnen Vestibulopathien sollten nachfolgende Behandlungsprinzipien und Empfehlungen berücksichtigt werden:
- **Funktionelle Schwindelsyndrome:** häufigste Form in der Praxis, diagnostisch herausfordernd, insbesondere bei episodischen und chronischen Vestibulopathien.
- **M. Menière:** mittelfristige Linderung durch komplikationsarme intratympanale Therapien; schwierige Abgrenzung von der vestibulären Migräne im Frühstadium.
- **Benigne paroxysmaler Lagerungsschwindel:** effektive Behandlungsmethoden verfügbar.
- **Akute unilaterale Vestibulopathien:** Frühzeitige Physiotherapie zur Förderung der vestibulären Kompensation ist essenziell.
- **Bilaterale Vestibulopathie und Schwindel im Alter:** Physiotherapie, Sturzprophylaxe.
- **Zoster oticus:** frühzeitige virostatische Therapie, Kortikoidbehandlung, Lokaltherapie
- **Seltene Syndrome:** Bogengangsdehiszenz und Vestibularisparoxysmie erfordern eine gezielte Befragung, Untersuchung und Anwendung der diagnostischen Kriterien.
- **Zervikogener Schwindel:** Konzept akzeptiert, Beweis steht aus, kein diagnostischer Nachweis möglich, objektiver Nystagmusnachweis erforderlich. Hypothesen zu Ursachen existieren.
- **Traumatische Perilymphfistel:** Frühzeitige Intervention verbessert die Prognose bei „Schwindel“.
- **Steuern von Kraftfahrzeugen**: geregelte Fahreignung: „BAST-Kriterien“.
- **Andere traumatische Schäden der otischen Kapsel**: je nach Schädigung ggf. Chirurgie, absteigende Kortikoidbehandlung, Antibiose.
- **Begriffsanpassungen:** Ersetzung veralteter Begriffe wie „Commotio labyrinthi“ und „Contusio labyrinthi“ für eine klare diagnostische Einordnung (nach ICD).
- **HNO-Begutachtung:** Objektive Bewertungskriterien ermöglichen eine präzise Zuordnung der Störung mit „Schwindel“ zum HNO-Fachbereich (periphere Vestibulopathien).

## Supplementary Information

Weiterführende Literatur
