# Supplementary material for: Otorhinolaryngologic diagnostics and treatment of vertigo syndromes
Source: HNO. 2025 Apr 7;73(9):628–49. [Article in German] doi: 10.1007/s00106-025-01592-6 (PMC12370841; doi:10.1007/s00106-025-01592-6)
Supplement: Supplementary file 1 — Weiterführende Literatur [file 106_2025_1592_MOESM1_ESM.pdf]

## Supplementary Material

## Weiterführende Literatur


1. Matzker J (1958) Fortschritte und Irrtümer der Vestibularisforschung zwischen Goltz (1879) und Bárány (1905). Laryngo-Rhinologie-Otologie 17;9:527-533
2. Feuerecker R, Habs M, Dieterich M, Strupp M (2015) Chronic subjective dizziness: Fewer symptoms in the early morning--a comparison with bilateral vestibulopathy and downbeat nystagmus syndrome. J Vestib Res 25(2):67-72
3. Watzlawick P, Janet H, Beavin DD (2000) Menschliche Kommunikation. Formen, Störungen, Paradoxien. Huber, Bern
4. Scharff JM (2020). Psychoanalyse und Zwischenleiblichkeit. Klinisch-propädeutisches Seminar. Brandes & Apsel, Frankfurt am Main
5. Hausteiner-Wiehle C, Henningsen P (2015) Kein Befund und trotzdem krank? Schattauer, Stuttgart
6. Weiland A, Blankenstein AH, Van Saase JL, Van der Molen HT, Jacobs ME, Abels DC, Köse N, Van Dulmen S, Vernhout RM, Arends LR (2015) Training Medical Specialists to Communicate Better with Patients with Medically Unexplained Physical Symptoms (MUPS). A Randomized, Controlled Trial. PLoS One Sep 18;10(9)
7. Popkirov S (2020). Funktionelle neurologische Störungen. Erkennen, verstehen, behandeln. Springer, Heidelberg
8. Schulz von Thun F (1981) Die Anatomie einer Nachricht. In: Schulz von Thun F (Hrsg) Miteinander Reden. Band 1: Störungen und Klärungen. Reinbek, Hamburg 1981, S 25–30
9. Jacobson GP, Newman CW (1990) The development of the Dizziness Handicap Inventory. Arch Otolaryngol Head Neck Surg 116:424–427
10. Kurre A, Christel JAW, Bastiaenen CHG, Gloor-Juzi T, Straumann D, de Bruin ED (2009) Translation, cross-cultural adaptation and reliability of the German version of the Dizziness Handicap Inventory. Otol Neurotol 30(3):359–367
11. Duong Dinh TA, Wittenborn J Westhofen M (2022). The Dizziness Handicap Inventory for quality control in the treatment of vestibular dysfunction. HNO Jan;70(1):19-23.
12. Volz-Sidiropoulou E, Takahama J, Gauggel S, Westhofen M (2010) The ‚Dizziness Handicap Inventory‘: initial psychometric evaluation of the German version. Laryngorhinootologie 89:418–423
13. Takahama JA, Volz-Sidiropoulou E, Westhofen M, Gauggel S (2010) Korrelation von "Dizziness Handicap Inventory" (DHI) Untersuchungsergebnissen in der Schwindeldiagnostik. Meeting Abstract. https://www.egms.de/static/en/meetings/hnod2010/10hnod456.shtml Zugegriffen: 13.11.2024
14. Tschan R, Wiltink J, Best C, Bense S, Dieterich M, Beutel ME, Eckhardt-Henn A. Validation of the German version of the Vertigo Symptom Scale (VSS) in patients with organic or somatoform dizziness and healthy controls. J Neurol 2008 Aug;255(8)
15. Gloor-Juzi T, Kurre A, Straumann D, de Bruin ED. Translation and validation of the vertigo symptom scale into German: A cultural adaption to a wider German-speaking population. BMC Ear Nose Throat Disord. 2012 Jul 2;12:
16. Yardley L, Masson E, Verschuur C, Haacke N, Luxon L: Symptoms, anxiety and handicap in dizzy patients: development of the vertigo symptom scale. J Psychosom Res 1992, 36 (8): 731-741. 10.1016/0022-3999(92)90131

17. Schott N. (2011). ABC-D. Activities-Specific Balance Confidence-Skala [Verfahrensdokumentation und Fragebogen]. In: Leibniz-Institut für Psychologie (ZPID) (Hrsg.), Open Test Archive. Trier: ZPID
18. https://www.testarchiv.eu/ Zugegriffen: 13.11.2024
19. Szmulewicz DJ, Waterston JA, Halmagyi GM, Mossman S, Chancellor AM, McLean CA, Storey E (2011) Sensory neuropathy as part of the cerebellar ataxia neuropathy vestibular areflexia syndrome. May 31;76(22):1903-10
20. Halmagyi GM, Kumar K, McGarvie LA (2022) The visually enhanced vestibulo-ocular reflex in CANVAS. J Neurol J Jan;269(1):490-49
21. Jahn K, Zwergal A, Schniepp R (2010) Gangstörungen im Alter. Deutsches Ärzteblatt 107;17:306-316
22. Tinetti ME (1986) Performance-oriented assessment of mobility problems in elderly patients. J Am Geriatr Soc. 1986 Feb;34(2):119-26
23. Podsiadlo D, Richardson S (1991) The timed "Up & Go": a test of basic functional mobility for frail elderly persons. J Am Geriatr Soc. 1991 Feb;39(2):142-148
24. Enright PL, Sherill DL (1998) Reference equations for the six minute walk test in healthy adults. Am J Respir Crit Care Med. Nov;158(5 Pt 1):1384-7
25. de Morton NA, Davidson M, Keating JL (2008) The de Morton Mobility Index (DEMMI): an essential health index for an ageing world. Health and Quality of Life Outcomes. 6:63
26. Braun T, Schulz RJ, Hoffmann M, Reinke J, Tofaute L, Urner C, Krämer H, Bock B, de Morton NA, Grüneberg C (2015) Die deutsche Version des De Morton Mobility Index (DEMMI) – Erste klinische Ergebnisse aus dem Prozess der interkulturellen Adaptation eines Mobilitätstests. Zeitschrift für Gerontologie & Geriatrie. 48 (2):154-163
27. Halmagyi GM, Curthoys IS (1988) A clinical sign of canal paresis. Arch Neurol; 45:737-739
28. Colebatch JG, Halmagyi GM (1992) Vestibular evoked potentials in human neck muscles before and after unilateral vestibular deafferentation. 42:1635-1636
29. Todd NP, Curthoys IS, Aw ST et al. (2004) Vestibular evoked ocular responses to air- (AC) and bone-conducted (BC) sound I: eye movements and timing in relation to vestibular evoked peri-ocular potentials (VEPP). J Vestib Res 14:123–124
30. Todd NP, Rosengren SM, Colebatch JG (2004). Vestibular evoked ocular responses to air-(AC) and bone-conducted (BC) sound II: a neuroanatomical and physiological interpretation of AC-OVEMPs. J Vestib Res; 14:215–216
31. Ulmer E, Chays A (2005) Curthoys and Halmagyi Head Impulse test: an analytical device. Ann Otolaryngol Chir Cervicofac 2005;122: 84-90
32. Bartl K, Lehnen N, Kohlbecher S, Schneider E (2009) Head impulse testing using video-oculography. Ann NY Acad Sci 1164:331–333
33. Schneider E, Villgrattner T, Vockeroth J et al (2009) EyeSeeCam: an eye movement-driven head camera for the examination of natural visual exploration. Ann NY Acad Sci 1164:461–467
34. Weber KP, MacDougall HG, Halmagyi GM et al (2009). Impulsive testing of semicircular-canal function using video-oculography. Ann NY Acad Sci 1164:486–491
35. MacDougall HG, McGarvie LA, Halmagyi GM, Curthoys IS, Weber KP (2013) The video Head Impulse Test (vHIT) detects vertical semicircular canal dysfunction. PLoS One 22:e61488
36. MacDougall HG, McGarvie LA, Halmagyi GM, Curthoys IS, Weber KP (2013) Application of the video head impulse test to detect vertical semicircular canal dysfunction. Otol Neurotol 34:974-979
37. Blödow A, Pannasch S, Walther LE (2013). Detection of isolated covert saccades with the video head impulse test in peripheral vestibular disorders. Auris Nasus Larynx 40:348-351

38. Walther LE, Cebulla M (2015) Tips & Tricks: dynamic frequency analysis of otolith function using vestibular evoked myogenic potentials (VEMP)]. Laryngorhinootologie 94:153-155
39. Walther LE, Cebulla M. Band limited chirp stimulation in vestibular evoked myogenic potentials. Eur Arch Otorhinolaryngol 2016; DOI: 10.1007/s00405-015-3888-y [in press]
40. Murofushi T Clinical application of vestibular evoked myogenic potential (VEMP). Auris Nasus Larynx 2016; 43: 367-376
41. Govender S, Fernando T, Dennis DL et al. Properties of 500Hz air- and bone-conducted vestibular evoked myogenic potentials (VEMPs) in superior canal dehiscence. Clin Neurophysiol 2016; 127: 2522-2531
42. Jacobson GP, McCaslin DL, Piker EG et al (2011) Patterns of abnormality in cVEMP, oVEMP, and caloric tests may provide topological information about vestibular impairment. Am Acad Audiol 22: 601-611
43. Walther LE, Brusis T (2013) From the expert's office: Current diagnosis and assessment of vestibular receptor function].Laryngorhinootologie. Mar;92(3):189-92
44. Tarnutzer AA, Christopher J Bockisch CJ, Buffone E, Weber KP (2020) Vestibular mapping in patients with unilateral peripheral-vestibular deficits. Neurology 1;95(22)
45. Walther LE, Brusis T, Meister E, Blödow A (2023). Aus der Gutachtenpraxis: Zeitgemäße HNO-Begutachtung bei peripheren Vestibulopathien. Teil 2: Vorschläge für neue Bewertungskriterien. Laryngorhinootologie. 2023 May;102(5):373-380
46. Edlow JA, Carpenter C, Akhter M, Khoujah D, Marcolini E, Meurer WJ, Morrill D, Naples JG, Ohle R, Omron R, Sharif S, Siket M, Upadhye S, E Silva LOJ, Sundberg E, Tartt K, Vanni S, Newman-Toker DE, Bellolio F (2023). Guidelines for reasonable and appropriate care in the emergency department 3 (GRACE-3): Acute dizziness and vertigo in the emergency department. Acad Emerg Med. 2May;30(5):442-486
47. Mantokoudis G, Saber Tehrani AS, Wozniak A, Eibenberger K, Kattah JC, Guede CI, Zee DS, Newman-Toker DE (2015). VOR gain by head impulse video-oculography differentiates acute vestibular neuritis from stroke. Otol Neurotol. 2015 Mar;36(3):457-65
48. Tarnutzer AA, Gold D, Wang Z, Robinson KA, Kattah JC, Mantokoudis G, Saber Tehrani AS, Zee DS Edlow JA, Newman-Toker DE (2023) Impact of Clinician Training Background and Stroke Location on Bedside Diagnostic Test Accuracy in the Acute Vestibular Syndrome - A Meta-Analysis. Ann Neurol Aug;94(2):295-308.
49. Tarnutzer AA, Edlow JA (2023) Bedside Testing in Acute Vestibular Syndrome-Evaluating HINTS Plus and Beyond-A Critical Review. Audiol Res Sep 1;13(5):670-685
50. Johnston SC, Rothwell PM, Nguyen-Huynh MN, Giles MF, Elkins JS, Bernstein AL, et al. (2007) Validation and refinement of scores to predict very early stroke risk after transient ischaemic attack. Lancet 369:283–292
51. Kattah JC, Talkad AV, Wang DZ et al. HINTS to diagnose stroke in the acute vestibular syndrome: three-step bedside oculomotor examination more sensitive than early MRI diffusion-weighted imaging. Stroke 2009; 40: 3504-3510
52. Korda A, Wimmer W, Zamaro E, Wagner F, Sauter TC, Caversaccio MD, Mantokoudis G (2022) Videooculography “HINTS” in Acute Vestibular Syndrome: A Prospective Study. Front. Neurol. Jul 12:13:920357
53. Sauvaget E, Kici S, Petelle B, Kania R, Chabriat H, Herman P, Tran Ba Huy P (2004) Vertebrobasilar occlusive disorders presenting as sudden sensorineural hearing loss. Laryngoscope Feb;114(2):327-32
54. Chang CF, Kuo YL, Chen, SP, Wang MC, Liao WH, Tu TY, Shiao AS (2013) Relationship between idiopathic sudden sensorineural hearing loss and subsequent stroke. Laryngoscope

Apr;123(4):1011-5
55. Zhong Y, Li H, Liu G, Liu J, Mo JJ, Zhao X, Ju Y (2023) Early detection of stroke at the sudden sensorineural hearing loss stage. Front Neurol. 2023 Nov 1:14:1293102
56. Yacovino DY Akly MP, Luis L, Zee DS (2018) The Floccular Syndrome: Dynamic Changes in Eye Movements and Vestibulo-ocular Reflex in Isolated Infarction of the Cerebellar Flocculus.Cerebellum Apr;17(2):122-131.
57. Gottlieb M, Peksa GD, Carlson JN (2923) Head impulse, nystagmus, and test of skew examination for diagnosing central causes of acute vestibular syndrome. Cochrane Database Syst Rev. 2023 Nov 2;11(11):CD015089
58. Tang B, Jiang W, Zhang C, Tan H, Luo M, He Y, Yu X (2024) Effect of public square dancing combined with serotonin reuptake inhibitors on persistent postural-perceptual dizziness (PPPD) in middle-aged and older women. J Vestib Res 34(1):63-72
59. Teh CS, Abdullah NA, Kamaruddin NR, Mohd Judi KB, Fadzilah I, Zainun Z, Prepageran N (2023) Home-based Vestibular Rehabilitation: A Feasible and Effective Therapy for Persistent Postural Perceptual Dizziness (A Pilot Study). N. Ann Otol Rhinol Laryngol. 2023 May;132(5):566-57
60. Axer H, Finn S, Wassermann A, Guntinas--Lichius O, Klinger CM, Witte OW. Multimodal treatment of persistent postural-perceptual dizziness. Brain Behav Dec;10(12):e01864
61. Suica Z, Behrendt F, Ziller C, Gäumann S, Schädler S, Hilfiker R, Parmar K, Gerth HU, Bonati LH, Schuster-Amft C (2024) Comparative effectiveness of non- pharmacological treatments in patients with persistent postural-perceptual dizziness: a systematic review and effect sizes analyses. Front Neurol 12;15:1426566
62. Walther LE, Blödow A, Buder J, Kniep R (2014) Principles of calcite dissolution in human and artificial otoconia. PLoS One Jul 21;9(7):e102516
63. Walther LE, Blödow A (2020) Current aspects of vertigo and dizziness in advanced age. HNO Mar;68(3):191-198
64. Walther LE, Wenzel A, Buder J, Bloching MB, Kniep R, Blödow A (2014). Detection of human utricular otoconia degeneration in vital specimen and implications for benign paroxysmal positional vertigo. Eur Arch Otorhinolaryngol Dec;271(12):3133-8
65. Walther LE (2016) Otoconia: Current aspects of research. HNO Oct;64(10):767-76
66. Walther LE, Westhofen M (2007) Presbyvertigo-aging of otoconia and vestibular sensory cells. J Vestib Res ;17(2-3):89-92
67. Andrade LR, Lins U, Farina M, Kachar B, Thalmann R (2012) Immunogold TEM of otoconin 90 and otolin - relevance to mineralization of otoconia, and pathogenesis of benign positional vertigo.Hear Res Oct;292(1-2):14-25
68. Fan Z, Hu Z, Han W, Lu X, Liu X, Zhou M, Yan W, Wu Y (2022) High Serum Levels of Otolin-1 in Patients With Benign Paroxysmal Positional Vertigo Predict Recurrence. Front Neurol Mar 14;13:841677
69. Kao WT, Parnes LS, Chole RA (2017) Otoconia and otolithic membrane fragments within the posterior semicircular canal in benign paroxysmal positional vertigo. Laryngoscope. Mar;127(3):709-714
70. Bronstein A, Lempert T. Schwindel. Ein praktischer Leitfaden zur Diagnose und Therapie 2. Auflage, Schattauer, Stuttgart
71. Kim JM, Lee SH, Cho SH, Kang KW, Choi KH, Nam TS, Kim JT, Choi SM, Park MS, Kim BC, Kim MK (2021) Cerebellar infarction presenting with isolated positional vertigo: differentiating factors for benign paroxysmal positional vertigo. Neurol Sci 42(3):1045-1052
72. https://content.iospress.com/articles/journal-of-vestibular-research/ves553. Zugegriffen am: 13.11.2024

73. von Brevern M, Seelig T, Neuhauser H, Lempert T. Benign paroxysmal positional vertigo predominantly affects the right labyrinth. J Neurol Neurosurg Psychiatry 4 Oct;75(10):1487-8
74. Kim HJ, Park J, Kim JS (2021) Update on benign paroxysmal positional vertigo. J Neurol. May;268(5):1995-2000
75. Brandt T, Steddin S (1993) Current view of the mechanism of benign paroxysmal positioning vertigo: cupulolithiasis or canalolithiasis? J Vestib Res 3(4):373-82
76. Schuknecht HF (1969) HF Cupulolithiasis. Arch Otolaryngol 90(6):765-78
77. Epley JM (1992) The canalith repositioning procedure: for treatment of benign paroxysmal positional vertigo. Otolaryngol Head Neck Surg 107:399– 404
78. Halmágyi GM, Akdal G, Welgampola MS, Wang C (2023) Neurological update: neuro-otology 2023. J Neurol 270(12):6170-6192
79. Shah MU, Lotterman S, Roberts D, Eisen M (2019) Smartphone telemedical emergency department consults for screening of nonacute dizziness. Laryngoscope 129(2):466-469
80. Melliti A, van de Berg M, van de Berg R (2023) Capturing nystagmus during vertigo attacks using a smartphone: adherence, characteristics, pearls and pitfalls. J Neurol 270(12):6044-6056
81. Choi JY, Glasauer S, Kim JH, Zee DS, Kim JS (2018) Characteristics and mechanism of apogeotropic central positional nystagmus Brain 1;141(3):762-775
82. Kwon E, Jeong HS, Jeong SH, Kim HJ, Kim JS (2014) Central paroxysmal positional nystagmus mimicking posterior canal benign paroxysmal positional vertigo in pontine infarction: a case report and literature review. J Neurol 271(6):3672-3677
83. Mun SB, Kim YJ, Lee JH, Han GC, Cho SH, Jin S, Kim KG (2024). Deep Learning-Based Nystagmus Detection for BPPV-Diagnosis. Sensors (Basel) 26;24(11):3417
84. Gufoni M, Mastrosimone L, Di Nasso F (1998) Repositioning maneuver in benign paroxysmal vertigo of horizontal semicircular canal. Acta Otorhinolaryngol Ital 18(6):363–367
85. Hilton MP, Pinder DK. The Epley (canalith repositioning) manoeuvre for benign paroxysmal positional vertigo (2014). Cochrane Database Syst Rev 8;2014(12):CD003162
86. Li W, Sun J, Zhao Z, Xu J, Wang H, Ding R, Zhang Y (2023) Efficacy of Epley's maneuver plus betahistine in the management of PC-BPPV: A systematic review and meta-analysis. Medicine (Baltimore) 31;102(13):e33421
87. Zhang X, Qian X, Lu L, Chen J, Liu J, Lin C, Gao X (2017) Effects of Semont maneuver on benign paroxysmal positional vertigo: a meta-analysis. Acta Otolaryngol 137(1):63-70
88. Maas BDPJ, van der Zaag-Loonen HJ, van Benthem PPG, Bruintjes TD (2020). Effectiveness of Canal Occlusion for Intractable Posterior Canal Benign Paroxysmal Positional Vertigo: A Systematic Review. Otolaryngol Head Neck Surg 162(1):40-49
89. Wang XY, Ji B, Yong J, Liu LZ (2013) Efficacy of the modified Epley maneuver versus modified Semont maneuver for posterior canal benign paroxysmal positional vertigo: a meta-analysis]. Zhonghua Er Bi Yan Hou Tou Jing Wai Ke Za Zhi 48(8):634-9
90. Hunt WT, Zimmermann EF, Hilton MP (2012) Modifications of the Epley (canalith repositioning) manoeuvre for posterior canal benign paroxysmal positional vertigo (BPPV). Cochrane Database Syst Rev 18;2012(4):CD008675
91. Fu W, Han J, Chang N, Wei D, Bai Y, Wang Y, He F, Wang X (2020). Immediate efficacy of Gufoni maneuver for horizontal canal benign paroxysmal positional vertigo (HC-BPPV): a meta-analysis. Auris Nasus Larynx Feb;47(1):48-54
123a Anagnostou E, Kouzi I, Spengos K (2015) Diagnosis and Treatment of Anterior-Canal Benign Paroxysmal Positional Vertigo: A Systematic Review. J Clin Neurol 11(3):262-7
92. Yang Z, Li J, Zhu Z, He J, Wei X, Xie M (2021). Effect of vitamin D supplementation on benign paroxysmal positional vertigo recurrence: A meta-analysis. Sci Prog. 104(2):368504211024569.

93. Jeong SH, Lee SU, Kim JS (2022) Prevention of recurrent benign paroxysmal positional vertigo with vitamin D supplementation: a meta-analysis. J Neurol 269(2):619-626
94. Wood H, Kluk K, BinKhamis G (2024). Association between vitamin D deficiency and benign paroxysmal positional vertigo (BPPV) incidence and recurrence: a systematic review and meta-analysis. BMJ Open 22;14(4):e077986
95. Sharif S, Khoujah D, Greer A, Naples JG, Upadhye S, Edlow JA (2023) Vestibular suppressants for benign paroxysmal positional vertigo: A systematic review and meta-analysis of randomized controlled trials. Acad Emerg Med 30(5):541-551
96. Bulğurcu S, Baz E, Güleryüz S, Erkul E, Çekin E (2022). Effect of applying head-shaking maneuver before Epley maneuver in BPPV. Braz J Otorhinolaryngol 88(6):932-936
97. Mandalà M, Califano L, Casani AP, Faralli M, Marcelli V, Neri G, Pecci R, Scasso F, Scotto di Santillo L, Vannucchi P, Giannoni B, Dasgupta S, Bindi I, Salerni L, Nuti D (2021) Double-Blind Randomized Trial on the Efficacy of the Forced Prolonged Position for Treatment of Lateral Canal Benign Paroxysmal Positional Vertigo. Laryngoscope. 131(4):E1296-E1300
98. Giannoni B, Pecci R, Pollastri F, Mininni S, Licci G, Santimone R, Di Giustino F, Mandalà M (2023). Treating benign paroxysmal positional vertigo of the lateral semicircular canal with a shortened forced position. Front Neurol 6;14:1153491
99. Han K, Lee J, Shin JE, Kim CH (2024) Treatment Efficacy of Forced Prolonged Position After Cupulolith Repositioning Maneuver in Apogeotropic HSCC BPPV. Ear Nose Throat J Apr;103(4)
100. Li Y, Smith RM, Whitney SL, Seemungal BM, Ellmers TJ (2023). We should be screening for benign paroxysmal positional vertigo (BPPV) in all older adults at risk of falling: a commentary on the World Falls Guidelines. Age Ageing 2;52(11)
101. Ribeiro KF, Oliveira BS, Freitas RV, Ferreira LM, Deshpande N, Guerra RO (2017). Effectiveness of Otolith Repositioning Maneuvers and Vestibular Rehabilitation exercises in elderly people with Benign Paroxysmal Positional Vertigo: a systematic review. Braz J Otorhinolaryngol. 2017 29;84(1):109–18
102. Sayin I, Koç RH, Temirbekov D, Gunes S, Cirak M, Yazici ZM (2022). Betahistine add-on therapy for treatment of subjects with posterior benign paroxysmal positional vertigo: a randomized controlled trial. Braz J Otorhinolaryngol 88(3):421-426
103. Cavaliere M, Mottola G, Iemma M. Benign paroxysmal positional vertigo: a study of two manoeuvres with and without betahistine (2005). Acta Otorhinolaryngol Ital. 25(2):107-12
104. Li W, Sun J, Zhao Z, Xu J, Wang H, Ding R, Zhang Y. Efficacy of Epley's maneuver plus betahistine in the management of PC-BPPV: A systematic review and meta-analysis (2023). Medicine (Baltimore). 31;102(13):e33421
105. Brandt T, Huppert D, Hecht J, Karch C, Strupp M (2006). Benign paroxysmal positioning vertigo: a long-term follow-up (6-17 years) of 125 patients. Acta Otolaryngol 126(2):160-3
106. Pérez P, Franco V, Cuesta P, Aldama P, Alvarez MJ, Méndez JC (2012) Recurrence of benign paroxysmal positional vertigo. Otol Neurotol 33(3):437-43
107. Chen J, Zhang S, Cui K, Liu C (2021) Risk factors for benign paroxysmal positional vertigo recurrence: a systematic review and meta-analysis J Neurol 268(11):4117-4127
108. Li S, Wang Z, Liu Y, Cao J, Zheng H, Jing Y, Han L, Ma X, Xia R, Yu L (2022). Risk Factors for the Recurrence of Benign Paroxysmal Positional Vertigo: A Systematic Review and Meta-Analysis. Ear Nose Throat J 101(3):NP112-NP134
109. https://youtu.be/KLt2LtISPmQ (in 141). Zugegriffen: 13.11.2024
110. Martín-Sierra C, Gallego-Martinez A, Requena T, Frejo L, Batuecas-Caletrío A, Lopez-Escamez JA (2017). Variable expressivity and genetic heterogeneity involving DPT and SEMA3D genes in autosomal dominant familial Ménière disease (2017). Eur J Hum Genet 25:200-207

111. Phillips J, Murdin L, Khondoker M, Grant K, Shepstone L, Sims E, Rea P, Harcourt J (2024) Cluster Analysis to Identify Clinical Subtypes of Ménière's Disease. Laryngoscope 134(7):3286-3292
112. Hoskin JL (2022). Ménière's disease: new guidelines, subtypes, imaging, and more. Curr Opin Neurol 1;35(1):90-97
113. Basura GJ, Adams ME, Monfared A, Schwartz SR, Antonelli PJ, Burkard R, Bush ML, Bykowski J, Colandrea M, Derebery J, Kelly EA, Kerber KA, Koopman CF, Kuch AA, Marcolini E, McKinnon BJ, Ruckenstein MJ, Valenzuela CV, Vosooney A, Walsh SA, Nnacheta LC, Dhepyasuwan N, Buchanan EM (2020) Clinical Practice Guideline: Ménière's Disease. Otolaryngol Head Neck Surg 162(2_suppl):S1-S55
114. Gürkov, R., Hornibrook, J (2018). On the classification of hydropic ear disease (Menière's disease). HNO 66, 455–463
115. Eliezer M, Attyé A, Toupet M, Hautefort C (2021) Imaging of endolymphatic hydrops: A comprehensive update in primary and secondary hydropic ear disease. J Vestib Res 31(4):261-268
116. Guajardo-Vergara C, Suárez-Vega V, Dominguez P, Manrique-Huarte R, Arbizu L, Pérez-Fernández N (2022). Endolymphatic hydrops in the unaffected ear of patients with unilateral Ménière's disease. Eur Arch Otorhinolaryngol 279(12):5591-5600
117. van der Lubbe MFJA, Vaidyanathan A, Van Rompaey V, Postma AA, Bruintjes TD, Kimenai DM, Lambin P, van Hoof M, van de Berg R (2020). The "hype" of hydrops in classifying vestibular disorders: a narrative review. J Neurol 267(Suppl 1):197-211
118. Sun W, Guo P, Ren T, Wang W. Magnetic resonance imaging of intratympanic gadolinium helps differentiate vestibular migraine from Ménière disease. Laryngoscope. 2017 Oct;127(10):2382-2388
119. Liu Y, Pyykkö I, Naganawa S, Marques P, Gürkov R, Yang J, Duan M (2022). Consensus on MR-Imaging of Endolymphatic Hydrops in Patients With Suspected Hydropic Ear Disease (Meniere). Front Surg 28;9:874971.
120. Radtke A, von Brevern M, Neuhauser H, Hottenrott T, Lempert T (2012). Vestibular migraine: long-term follow-up of clinical symptoms and vestibulo-cochlear findings. Neurology 9;79(15):1607-14
121. Neff BA, Staab JP, Eggers SD, Carlson ML, Schmitt WR, Van Abel KM, Worthington DK, Beatty CW, Driscoll CL, Shepard NT (2012). Auditory and vestibular symptoms and chronic subjective dizziness in patients with Ménière's disease, vestibular migraine, and Ménière's disease with concomitant vestibular migraine. Otol Neurotol 33(7):1235-44
122. Chen JY, Guo ZQ, Wang J, Liu D, Tian E, Guo JQ, Kong WJ, Zhang SL (2023) Vestibular migraine or Meniere's disease: a diagnostic dilemma. J Neurol 270(4):1955-1968
123. https://content.iospress.com/articles/journal-of-vestibular-research/ves00453 Zugegriffen: 13.11.2024
124. Headache Classification Committee of the International Headache Society (IHS) 2013 The International Classification of Headache Disorders, 3rd edition (beta version). Cephalalgia. 33(9):629-808 update: https://ichd-3.org (siehe Appendix): Zugegriffen 13.11.2024
125. McGarvie LA, Curthoys IS, MacDougall HG, Halmagyi GM (2015). What does the head impulse test versus caloric dissociation reveal about vestibular dysfunction in Ménière's disease? Ann N Y Acad Sci 1343:58-62
126. Blödow A, Heinze M, Bloching MB, von Brevern M, Radtke A, Lempert T. (2014) Caloric stimulation and video-head impulse testing in Ménière's disease and vestibular migraine. Acta Otolaryngol 134(12):1239-44.
127. Balayeva F, Kirazlı G, Celebisoy N (2023) Vestibular test results in patients with vestibular

migraine and Meniere's Disease. Acta Otolaryngol 143(6):471-475

128. Kaçan M, Kirazlı G, Balayeva F, Celebisoy N (2023). Recurrent Vestibulopathy: Comparison of Vestibular Test Results with Ménière's Disease and Vestibular Migraine. Audiol Neurootol 28(2):116-127
129. Zhu Y, McPherson J, Beatty C, Driscoll C, Neff B, Eggers S, Shepard NT (2014) Cervical VEMP threshold response curve in the identification of Ménière's disease. J Am Acad Audiol. 25(3):278-88
130. Brandt T, Huppert T, Hüfner K, Zingler VC, Dieterich M, Strupp M (2010). Long-term course and relapses of vestibular and balance disorders. Restor Neurol Neurosci 28(1):69-82
131. Magnan J, Özgirgin ON, Trabalzini F, Lacour M, Escamez AL, Magnusson M, Güneri EA, Guyot JP, Nuti D, Mandalà M (2018) European Position Statement on Diagnosis, and Treatment of Meniere's Disease. J Int Adv Otol 14(2):317-321
132. Basura GJ, Adams ME, Monfared A, Schwartz SR, Antonelli PJ, Burkard R, Bush ML, Bykowski J, Colandrea M, Derebery J, Kelly EA, Kerber KA, Koopman CF, Kuch AA, Marcolini E, McKinnon BJ, Ruckenstein MJ, Valenzuela CV, Vosooney A, Walsh SA, Nnacheta LC, Dhepyasuwan N, Buchanan EM (2020) Clinical Practice Guideline: Ménière's Disease. Otolaryngol Head Neck Surg 162(2_suppl):S1-S55.6
133. Ahmadzai N, Cheng W, Kilty S, Esmaeilisaraji L, Wolfe D, Bonaparte J, Schramm D, Fitzpatrick E, Lin V, Skidmore B, Hutton B. Pharmacologic and surgical therapies for patients with Meniere's disease: A systematic review and network meta-analysis. PLoS One. 2020 Sep 1;15(9):e0237523
134. Szott FA, Westhofen M, Hackenberg S (2023). Is endolymphatic sac surgery an efficient treatment of Menière's disease patients? A systematic literature search and meta-analysis. Eur Arch Otorhinolaryngol. ar;280(3):1119-1128
135. Taniguchi AN, Sutton SR, Mills JF, Nguyen SA, Rizk HG, Meyer TA, Nguyen JP, Lambert PR (2024) Placebo effect in randomized controlled trials for Meniere's disease: A meta-analysis. Am J Otolaryngol 45(2):104178. doi: 10.1016/j.amjoto.2023
136. Hessel FP (2024) Stufentherapiekonzept bei Morbus Menière: Eine retrospektive Analyse. Inaugural-Dissertation zur Erlangung des Doktorgrades der gesamten Humanmedizin dem Fachbereich Medizin der Philipps-Universität Marburg. Inaugural-Dissertation
137. Schaaf H, Haid CT. Reaktiver psychogener Schwindel bei Morbus Menière (2993) Dtsch Arztebl 100(13): A-853 / B-720 / C-673
138. Hussain K, Murdin L, Schilder AG (2018). Restriction of salt, caffeine and alcohol intake for the treatment of Ménière's disease or syndrome. Cochrane Database Syst Rev 31;12(12):CD012173
139. van Sonsbeek S, Pullens B, van Benthem PP (2015) Positive pressure therapy for Ménière's disease or syndrome. Cochrane Database Syst Rev 10;2015(3):CD008419.
140. Adrion C, Fischer CS, Wagner J, Gürkov R, Mansmann U, Strupp M (2016) BEMED Study Group. Efficacy and safety of betahistine treatment in patients with Meniere's disease: primary results of a long term, multicentre, double blind, randomised, placebo controlled, dose defining trial. BMJ 21;352:h6816
141. Ernst A, Schlattmann P, Waldfahrer F, Westhofen M (2017) Die Behandlung des M. Menière mit Betahistin: Kritische Anmerkungen zur BEMED-Studie. Laryngorhinootologie 96(8):519-521
142. Van Esch B, van der Zaag-Loonen H, Bruintjes T, van Benthem PP (2022) Betahistine in Ménière's Disease or Syndrome: A Systematic Review. Audiol Neurootol 27(1):1-33
143. Murdin L, Hussain K, Schilder AG (2016). Betahistine for symptoms of vertigo. Cochrane Database Syst Rev 21;2016(6)
144. Strupp M, Churchill GC, Naumann I, Mansmann U, Al Tawil A, Golentsova A, Goldschagg N (2023) Examination of betahistine bioavailability in combination with the monoamine oxidase

B inhibitor, selegiline, in humans-a non-randomized, single-sequence, two-period titration, open label single-center phase 1 study (PK-BeST) Front Neurol 18;14:1271640
145. Sanković-Babić S, Kosanović R, Ivanković Z, Babac S, Tatović M (2014) Intratympanic corticosteroid perfusion in the therapy of Meniere's disease. Srp Arh Celok Lek. 142(5-6):291-5
146. Patel M, Agarwal K, Arshad Q, Hariri M, Rea P, Seemungal BM, Golding JF, Harcourt JP, Bronstein AM (2016) Intratympanic methylprednisolone versus gentamicin in patients with unilateral Ménière's disease: a randomised, double-blind, comparative effectiveness trial. Lancet 3;388(10061):2753-2762
147. Albu S, Chirtes F, Trombitas V, Nagy A, Marceanu L, Babighian G, Trabalzini F (2015) Intratympanic dexamethasone versus high dosage of betahistine in the treatment of intractable unilateral Meniere disease. Am J Otolaryngol 36(2):205-9
148. Molnár A, Maihoub S, Tamás L, Szirmai Á (2019) Intratympanically administered steroid for progressive sensorineural hearing loss in Ménière's disease. Acta Otolaryngol 139(11):982-986
149. Phillips J, Mikulec AA, Robinson JM, Skarinsky D, Anderson JJ (2023) Efficacy of Intratympanic OTO-104 for the Treatment of Ménière's Disease: The Outcome of Three Randomized, Double-Blind, Placebo-Controlled Studies. Otol Neurotol 1;44(6):584-592.
150. van Esch BF, van der Zaag-Loonen H, Bruintjes T, Kuijpers T, van Benthem PPG (2022) Interventions for Menière's disease: an umbrella systematic review. BMJ Evid Based Med 27(4):235-245
151. Hao W, Yu H, Li H (2022) Effects of intratympanic gentamicin and intratympanic glucocorticoids in Ménière's disease: a network meta-analysis. J Neurol 269(1):72-86
152. de Amesti F, Santander MJ, Winter M (2022) Intratympanic gentamicin compared with placebo for Ménière's disease. Medwave 22;22(2):e8696
153. Li S, Pyykkö I, Zhang Q, Yang J, Duan M. Consensus on intratympanic drug delivery for Menière's disease. Eur Arch Otorhinolaryngol. 2022 Aug;279(8):3795-3799
154. Hao W, Yu H, Li H (2022) Effects of intratympanic gentamicin and intratympanic glucocorticoids in Ménière's disease: a network meta-analysis. J Neurol 269(1):72-86
155. Lange G, Mann W, Maurer J (2003) Intratympanic interval therapy of Menière disease with gentamicin with preserving cochlear function. HNO Nov;51(11):898-902
156. Faizal B, Rajan A (2022) Low Dose Intratympanic Gentamicin in Ménière's Disease. Indian J Otolaryngol Head Neck Surg 74(Suppl 1):320-325
157. Minor LB (1999) Intratympanic gentamicin for control of vertigo in Meniere's disease: vestibular signs that specify completion of therapy. Am J Otol 20(2):209-19.
158. Gode S, Celebisoy N, Akyuz A, Gulec F, Karapolat H, Bilgen C, Kirazli T (2011). Single-shot, low-dose intratympanic gentamicin in Ménière disease: role of vestibular-evoked myogenic potentials and caloric test in the prediction of outcome. Am J Otolaryngol 32(5):412-6
159. Salt AN, Gill RM, Plontke SK (2008) Dependence of hearing changes on the dose of intratympanically applied gentamicin: a meta-analysis using mathematical simulations of clinical drug delivery protocols. Laryngoscope 118(10):1793-800
160. Lyu Y, Zhang D, Li X, Han Y, Li Y, Wang J, Song Y, Kong L, Jian H, Fan Z, Wang H (2020) Dexamethasone protects the hearing of Meniere's disease patients after triple semicircular canal plugging. Acta Otolaryngol140(10):803-807
161. Berardino FD, Conte G, Turati F, Ferraroni M, Zanetti D. Cochlear implantation in Ménière's disease: a systematic review of literature and pooled analysis (2020) Int J Audiol 59(6):406-415
162. Selleck AM, Dillon M, Perkins E, Brown KD (2021) Cochlear Implantation in the Setting of Menière's Disease After Labyrinthectomy: A Meta-Analysis. Otol Neurotol1;42(8):e973-e979
163. Doobe G, Ernst A, Ramalingam R, Mittmann P, Todt I (2015) Simultaneous Labyrinthectomy

and Cochlear Implantation for Patients with Single-Sided Ménière's Disease and Profound Sensorineural Hearing Loss. Biomed Res Int: 457318. doi: 10.1155/2015/457318
164. Canzi P, Manfrin M, Perotti M, Aprile F, Quaglieri S, Rebecchi E, Locatelli G, Benazzo M (2016). Translabyrinthine vestibular neurectomy and simultaneous cochlear implant for Ménière's disease. Acta Neurochir (Wien) 159(1):123-130
165. Curthoys IS, Burgess AM, Manzari L (2020) The Evidence for Selective Loss of Otolithic Function. Semin Neurol 33-39. doi: 10.1055/s-0039-3402064
166. Büki B, Ward BK (2021). Length of the Narrow Bony Channels May Not be the Sole Cause of Differential Involvement of the Nerves in Vestibular Neuritis. Otol Neurotol. 2021 Aug 1;42(7):e918-e924.
167. Kamo T, Ogihara H, Azami M, Momosaki R, Fushiki H (2023) Effects of Early Vestibular Rehabilitation in Patients With Acute Vestibular Disorder: A Systematic Review and Meta-Analysis. Otol Neurotol 1;44(9):e641-e647
168. Lacour M, Lopez C, Thiry A, Tardivet L (2023) Vestibular rehabilitation improves spontaneous nystagmus normalization in patients with acute unilateral vestibulopathy. Front Rehabil Sci. 2023 May 31;4:1122301
169. Strupp M, Zingler VC, Arbusow V, Niklas D, Maag KP, Dieterich M, Bense S, Theil D, Jahn K, Brandt T (2004) Methylprednisolone, valacyclovir, or the combination for vestibular neuritis. N Engl J Med 22;351(4):354-61
170. Vestibular neuritis: Anti-vertigo drugs at most for three days!. MMW Fortschr Med. 2006 Jun 8;148(23):14. German
171. Scherer H (1996) Das Gleichgewicht. 2. Auflage 1996. Springer Verlag Berlin Heidelberg New York.
172. Helmchen C, Klinkenstein J, Machner B, Rambold H, Mohr C, Sander T (2009). Structural changes in the human brain following vestibular neuritis indicate central vestibular compensation. Ann N Y Acad Sci 1164:104-15
173. Lacour M, Helmchen C, Vidal PP et al. (2016). Vestibular compensation: the neuro-otologist's best friend. J Neurol 263: Suppl 1: S54-64
174. Lacour M, Dutheil S, Tighilet B, Lopez C, Borel L (2009). Tell me your vestibular deficit, and i'll tell you how you'll compensate. Ann N Y Acad Sci 1164:268-78
175. Yazdanshenas H, Ashouri A, Kaufman G (2016) Neurovestibular Compensation following Ototoxic Lesion and Labyrinthectomy. Int Arch Otorhinolaryngol;20(2):114-23
176. El Mahmoudi N, Laurent C, Péricat D, Watabe I, Lapotre A, Jacob PY, Tonetto A, Tighilet B, Sargolini F (2023) Long-lasting spatial memory deficits and impaired hippocampal plasticity following unilateral vestibular loss. Prog Neurobiol 223:102403
177. Walther LE, Brusis T, Meister E, Blödow A (2023). Aus der Gutachtenpraxis: Zeitgemäße HNO-Begutachtung bei peripheren Vestibulopathien. Teil 2: Vorschläge für neue Bewertungskriterien. Laryngorhinootologie. 2023 May;102(5):373-380. German.
178. Palla A, Straumann D (2004) Recovery of the high-acceleration vestibulo-ocular reflex after vestibular neuritis. J Assoc Res Otolaryngoll 5(4):427-35
179. Strupp M, Arbusow V (1998) Therapie bei Schwindel [Therapy of vertigo]. Dtsch Med Wochenschr. 1998 Sep 4;123(36):1041-5. German
180. Brantberg K, Löfqvist L (2007) Preserved vestibular evoked myogenic potentials (VEMP) in some patients with walking-induced oscillopsia due to bilateral vestibulopathy. J Vestib Res 17(1):33-8
181. Bürgin A, Bockisch CJ, Tarnutzer AA (2018) Precision of perceived direction of gravity in partial bilateral vestibulopathy correlates with residual utricular function. Clin Neurophysiol. 2018

May;129(5):934-945
182. Fujimoto C, Yagi M, Murofushi T (2019) Recent advances in idiopathic bilateral vestibulopathy: a literature review. Orphanet J Rare Dis Aug 19;14(1):202
183. Fujimoto C, Yagi M, Murofushi T (2019) Recent advances in idiopathic bilateral vestibulopathy: a literature review. Orphanet J Rare Dis Aug 19;14(1):202
184. Murofushi T, Monobe H, Ushio M (2023) Isolated bilateral posterior semicircular canal hypofunction: comparison with bilateral vestibulopathy. Acta Otolaryngol Aug;143(8):687-691
185. van Stiphout L, Lucieer F, Pleshkov M, Van Rompaey V, Widdershoven J, Guinand N, Pérez Fornos A, Kingma H, van de Berg R (2022) Bilateral vestibulopathy decreases self-motion perception. J Neurol Oct;269(10):5216-5228
186. van Stiphout L, Szmulewicz DJ, Guinand N, Fornos AP, Van Rompaey V, van de Berg R (2023) Bilateral vestibulopathy: a clinical update and proposed diagnostic algorithm. Front Neurol Dec 19;14:1308485
187. Gu H, Xu Y, Xu J, Zhuang J (2024) A rare case report of bilateral vestibulopathy due to otosyphilis. Medicine (Baltimore) May 17;103(20):e38149
188. Kim JG, Lee SU, Lee CN, Yu SW, Park KW, Kim JS (2020) Bilateral vestibulopathy as an early manifestation of systemic lupus erythematosus. J Neurol Jun;267(6):1855-1858
189. Elyoseph Z, Geisinger D, Zaltzman R, Mintz M, Gordon CR (2024) The vestibular symptomatology of Machado-Joseph Disease. J Vestib Res 34(2-3):159-167
190. Gürkov R, Manzari L, Blödow A, Wenzel A, Pavlovic D, Luis L (2018) Amiodarone-associated bilateral vestibulopathy. Eur Arch Otorhinolaryngol. 2018 Mar;275(3):823-825
191. Trache MC, Köntopf J, Stölzel K, Häußler SM (2024) Sudden Deafness and Vestibulopathy in a Patient with Antibody Treatment for Metastatic Lung Cancer. Laryngoscope Oct;134(10):4347-4350
192. Traschütz A, Heindl F, Bilal M, Hartmann AM, Dufke C, Riess O, Zwergal A, Rujescu D, Haack T, Synofzik M, Strupp M (2023) Frequency and Phenotype of RFC1 Repeat Expansions in Bilateral Vestibulopathy. Neurology Sep 5;101(10):e1001-e1013
193. Chow MR, Ayiotis AI, Schoo DP, Gimmon Y, Lane KE, Morris BJ, Rahman MA, Valentin NS, Boutros PJ, Bowditch SP, Ward BK, Sun DQ, Treviño Guajardo C, Schubert MC, Carey JP, Della Santina CC (2021) Posture, Gait, Quality of Life, and Hearing with a Vestibular Implant. N Engl J Med. 2021 Feb 11;384(6):521-532
194. Ren P, Zhang Q, Han P, Xu S, Xu C, Li Z, Yang J (2022) The working principle and prototype construction of the Chinese vestibular prosthesis. Lin Chuang Er Bi Yan Hou Tou Jing Wai Ke Za Zhi Sep;36(9):690-697
195. Schoo DP, Ward BK, Chow MR, Ayiotis AI, Fernández Brillet C, Boutros PJ, Lane KE, Lee CN, Morris BJ, Carey JP, Della Santina CC (2024) Vestibular Implant Surgery. Laryngoscope. 2024 Apr;134(4):1842-1846
196. Chow MR, Ayiotis AI, Schoo DP, Gimmon Y, Lane KE, Morris BJ, Rahman MA, Valentin NS, Boutros PJ, Bowditch SP, Ward BK, Sun DQ, Treviño Guajardo C, Schubert MC, Carey JP, Della Santina CC (2021) Posture, Gait, Quality of Life, and Hearing with a Vestibular Implant. N Engl J Med Feb 11;384(6):521-532
197. Avula S, Kumar S, Vadivu S, Kameswaran M (2024) Age dependent vestibulo-ocular reflex gain in video head impulse test among normal Indians. Bioinformation 31;20(5):520-527
198. Mossman B, Mossman S, Purdie G, Schneider E (2015) Age dependent normal horizontal VOR gain of head impulse test as measured with video-oculography. J Otolaryngol Head Neck Surg Jul 4;44(1):29
199. McGarvie LA, MacDougall HG, Halmagyi GM, Burgess AM, Weber KP, Curthoys IS (2015) The

Video Head Impulse Test (vHIT) of Semicircular Canal Function - Age-Dependent Normative Values of VOR Gain in Healthy Subjects. Front Neurol Jul 8;6:154
200. Pogson JM, Taylor RL, Bradshaw AP, McGarvie L, D'Souza M, Halmagyi GM, Welgampola MS (2019) The human vestibulo-ocular reflex and saccades: normal subjects and the effect of age. J Neurophysiol Jul 1;122(1):336-349
201. Mallinson AI, Longridge NS (2004) Caloric response does not decline with age. J Vestib Res 14(5):393-6
202. Ji L, Zhai S (2018) Aging and the peripheral vestibular system. J Otol 13(4):138-140. Erratum in: J Otol 2020 Dec;15(4):180
203. Rosa MS, Campagnoli M, Masnaghetti D, Taranto F, Pisani G, Garzaro M, Aluffi Valletti P (2023) Clinical and Prognostic Implications of Cervical and Ocular Vestibular Evoked Myogenic Potentials (cVEMP and oVEMP) in Benign Paroxysmal Positional Vertigo (BPPV): A Prospective Study. Audiol Res 12;13(5):700-709
204. Pauwels S, Casters L, Lemkens N, Lemmens W, Meijer K, Meyns P, van de Berg R, Spildooren J (2023) Gait and Falls in Benign Paroxysmal Positional Vertigo: A Systematic Review and Meta-analysis. J Neurol Phys Ther 1;47(3):127-138
205. Liao K, Walker MF, Joshi A, Reschke M, Leigh RJ (2008) Vestibulo-ocular responses to vertical translation in normal human subjects. Exp Brain Res. 2008 Mar;185(4):553-62
206. Srulijes K, Mack DJ, Klenk J, Schwickert L, Ihlen EA, Schwenk M, Lindemann U, Meyer M, Srijana KC, Hobert MA, Brockmann K, Wurster I, Pomper JK, Synofzik M, Schneider E, Ilg U, Berg D, Maetzler W, Becker C (2015) Association between vestibulo-ocular reflex suppression, balance, gait, and fall risk in ageing and neurodegenerative disease: protocol of a one-year prospective follow-up study. BMC Neurol 2015 Oct 9;15:192
207. Kennedy PGE, Mogensen TH, Cohrs RJ (2021) Recent Issues in Varicella-Zoster Virus Latency. Viruses 7;13(10)
208. https://www.rki.de/DE/Content/Kommissionen/STIKO/Empfehlungen/Aktuelles/Impfkalender.pdf?__blob=publicationFile Zugegriffen: 13.11.2024
209. https://www.rki.de/DE/Content/Infekt/EpidBull/Archiv/2018/Ausgaben/50_18.pdf?__blob=publicationFile Zugegriffen: 13.11.2024
210. Scotta MC, Paternina-de la Ossa R, Lumertz MS, Jones MH, Mattiello R, Pinto LA (2018) Early impact of universal varicella vaccination on childhood varicella and herpes zoster hospitalizations in Brazil. Vaccine. 2018 Jan 4;36(2):280-284
211. de Oliveira Gomes J, Gagliardi AM, Andriolo BN, Torloni MR, Andriolo RB, Puga MEDS, Canteiro Cruz E (2023) Vaccines for preventing herpes zoster in older adults. Cochrane Database Syst Rev. 2023 Oct 2;10(10):CD00885
212. Gross GE, Eisert L, Doerr HW, Fickenscher H, Knuf M, Maier P, Maschke M, Müller R, Pleyer U, Schäfer M, Sunderkötter C, Werner RN, Wutzler P, Nast A (2020) S2k-Leitlinie zur Diagnostik und Therapie des Zoster und der Postzosterneuralgie. J Dtsch Dermatol Ges. 18(1):55-79. German
213. Erskine N, Tran H, Levin L, Ulbricht C, Fingeroth J, Kiefe C, Goldberg RJ, Singh S (2017) A systematic review and meta-analysis on herpes zoster and the risk of cardiac and cerebrovascular events. PLoS One 27;12(7):e0181565
214. Uscategui T, Dorée C, Chamberlain IJ, Burton MJ (2008) Antiviral therapy for Ramsay Hunt syndrome (herpes zoster oticus with facial palsy) in adults. Cochrane Database Syst Rev. Oct 8;(4):CD006851
215. Minor LB, Solomon D, Zinreich JS, Zee DS (1998) Sound- and/or pressure-induced vertigo due to bone dehiscence of the superior semicircular canal. Arch Otolaryngol Head Neck Surg. 1998

Mar;124(3):249-58
216. Ward BK, Wenzel A, Ritzl EK, Gutierrez-Hernandez S, Della Santina CC, Minor LB, Carey JP (2013). Near-dehiscence: clinical findings in patients with thin bone over the superior semicircular canal. Otol Neurotol. 2013 Oct;34(8):1421-8
217. https://pmc.ncbi.nlm.nih.gov/articles/PMC9249274/pdf/ves-31-ves200004.pdf Zugegriffen: 13.11.2024
218. Carey JP, Minor LB, Nager GT (2000). Dehiscence or thinning of bone overlying the superior semicircular canal in a temporal bone survey. Arch Otolaryngol Head Neck Surg Feb;126(2):137-47
219. Lee JA, Liu YF, Nguyen SA, McRackan TR, Meyer TA, Rizk HG. (2020) Posterior Semicircular Canal Dehiscence: Case Series and Systematic Review. Otol Neurotol. 2020 Apr;41(4):511-521
220. Silverstein H, Van Ess MJ (2009) Complete round window niche occlusion for superior semicircular canal dehiscence syndrome: a minimally invasive approach. Ear Nose Throat J 2009 88(8):1042-56
221. Succar EF, Manickam PV, Wing S, Walter J, Greene JS, Azeredo WJ. Round window plugging in the treatment of superior semicircular canal dehiscence. Laryngoscope. 2018 Jun;128(6):1445-1452. doi: 10.1002/lary.26899. Epub 2017 Oct 9. PMID: 28990655.
222. Bunne M, Andersson H, Myhrum M (2022) Long-Term Outcomes of Round Window Reinforcement for Superior Semicircular Canal Dehiscence Syndrome. Otol Neurotol 1;43(6):709-716
223. Rommer PS, Wiest G, Kronnerwetter C, Zach H, Loader B, Elwischger K, Trattnig S (2015) 7-Tesla MRI demonstrates absence of structural lesions in patients with vestibular paroxysmia. Front Neuroanat 9;9:81
224. Cowen JE, Tehrani MS, Connor SE (2024) The association of vascular loops within the internal auditory meatus or contacting the vestibulo-cochlear nerve with audio-vestibular symptoms. A systematic review and meta-analysis. AJNR Am J Neuroradiol 4:ajnr.A8486
225. Kim CH, Choi KD (2021) Periodic Tinnitus and Direction-Changing Nystagmus in Vestibular Paroxysmia. J Clin Neurol. 2021 Jul;17(3):493-495
226. Sakaji K, Omodaka S, Kanamori M, Takai S, Sawada A, Suzuki J, Katori Y, Endo H (2024). Vestibular paroxysmia caused by a subarcuate artery: illustrative case. J Neurosurg Case Lessons. 2024 Sep 2;8(10):CASE24239
227. Xue H, Xiang W, Yu Y, Liu G, Chong Y, Zhou J (2018). Randomized trial of betahistine mesilate tablets as augmentation for oxcarbazepine and carbamazepine in treating vestibular paroxysmia. Drug Des Devel Ther. 2018 Apr 12;12:837-843
228. Hatano A, Rikitake M, Komori M, Irie T, Moriyama H (2009) Traumatic perilymphatic fistula with the luxation of the stapes into the vestibule. Auris Nasus Larynx 36(4):474–478
229. Rawal R, Zhao X, Lipson S, R Brodsky J (2021) Endoscopic Repair of Traumatic Perilymphatic Fistula in Children: A Case Series. J Int Adv Otol 17(2):182-185
230. Michel O (2024) *Gutachten in der HNO. Springer, Heidelberg*
231. Feldmann H, Brusis T (Hrsg) (2024*). Das Gutachten des HNO-Arztes. Thieme, Stuttgart*
232. Widder B et al (2024) Begutachtung nach gedecktem Schädel-Hirntrauma im Erwachsenenalter. AWMF-Leitlinienregister-Nr. 094-002. AWMF online
233. Walther LE, Hülse R, Lauer K, Wenzel A (2015) Current aspects of ototoxicity. Ototoxic substances and their effects]. HNO 63(4):315-24; quiz 325-6
234. Reid SA, Callister R, Katekar MG, Rivett DA (2014) Effects of cervical spine manual therapy on range of motion, head repositioning, and balance in participants with cervicogenic dizziness: a randomized controlled trial. Arch Phys Med Rehabil 95(9):1603-12

235. Reid SA, Callister R, Snodgrass SJ, Katekar MG, Rivett DA (2015) Manual therapy for cervicogenic dizziness: Long-term outcomes of a randomised trial. Man Ther (1):148-56
